# Reactive fluorescent probe for covalent membrane-anchoring: enabling real-time imaging of protein aggregation dynamics in live cells

**DOI:** 10.1039/d5sc07716h

**Published:** 2026-01-06

**Authors:** Hongbei Wei, Liren Xu, Ke Wei, Wenhai Bian, Yifan Wen, Wanyi Yu, Hui Zhang, Haiqiao Huang, Tony D. James, Xiaolong Sun

**Affiliations:** a The Key Laboratory of Biomedical Information Engineering of Ministry of Education, School of Life Science and Technology, Xi'an Jiaotong University No. 28 West Xianning Road Xi'an 710049 P. R. China x.l.sun86@xjtu.edu.cn huizhang@xjtu.edu.cn; b Department of Chemistry, University of Bath BA2 7AY Bath UK James@bath.ac.uk; c School of Chemistry and Chemical Engineering, Henan Normal University Xinxiang 453007 P. R. China; d Department of Urology, The Second Affiliated Hospital of Xi’an Jiaotong University No. 157 Xiwu Road, Xincheng District Xi’an 710004 China

## Abstract

Aberrant aggregation of membrane proteins is a pathological hallmark of various diseases, including neurodegenerative disorders and cancer. The visualization of membrane protein aggregation has emerged as an important approach for investigating protein structure and function and for studying disease mechanisms and therapeutic interventions. While significant progress has been made in modifying membrane proteins and studying related biological processes, membrane protein aggregation remains underexplored, largely due to the lack of simple and effective methods for directly labeling native proteins and tracking this process in real time. With this research, we present a fluorescent probe equipped with a membrane-anchoring unit and a covalent reactive moiety for visualizing membrane protein dynamics, which operates *via* a two-stage mechanism: first, rapid electrostatic interaction-mediated localization to the cell membrane, followed by chemoselective macrocyclization with thiol and amine groups on membrane proteins to form a fluorescent conjugate, whose emission is substantially enhanced due to restriction of twisted intramolecular charge transfer (TICT) within the confined microenvironment induced by protein aggregation. Leveraging this mechanism, the probe successfully reports membrane protein aggregation triggered by diverse stressors, such as redox imbalance and chemotherapeutic agents, while also capturing distinct membrane reorganization dynamics. With features of biocompatibility, wash-free performance, and long-term membrane retention, this probe provides an alternative tool for evaluating the complex structural dynamics of membrane proteins and offers potential for developing targeted therapeutic strategies.

## Abstract 2

A probe for non-covalent-to-covalent labeling of membrane proteins through macrocyclization and real-time fluorescence monitoring of protein aggregation.

## Graphical abstract

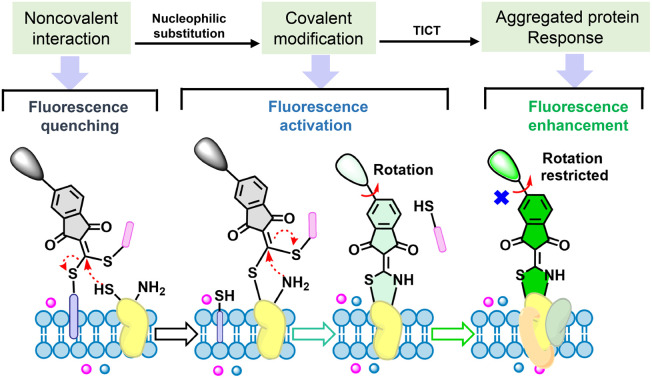

## Introduction

Membrane proteins are essential regulators of cellular interactions with the extracellular environment, playing pivotal roles in physiological processes including cell proliferation and differentiation.^1^ However, abnormal aggregation of membrane proteins (whether through misfolding or excessive clustering) is often linked to the pathological mechanisms of several major diseases, such as the formation of Aβ oligomers in Alzheimer’ disease and the clustering of EGFR receptors in cancer.^2–7^ Therefore, elucidating the real-time dynamics of membrane protein aggregation is crucial for understanding disease mechanisms and developing targeted therapeutic strategies.^8^

**Fig. 1:**
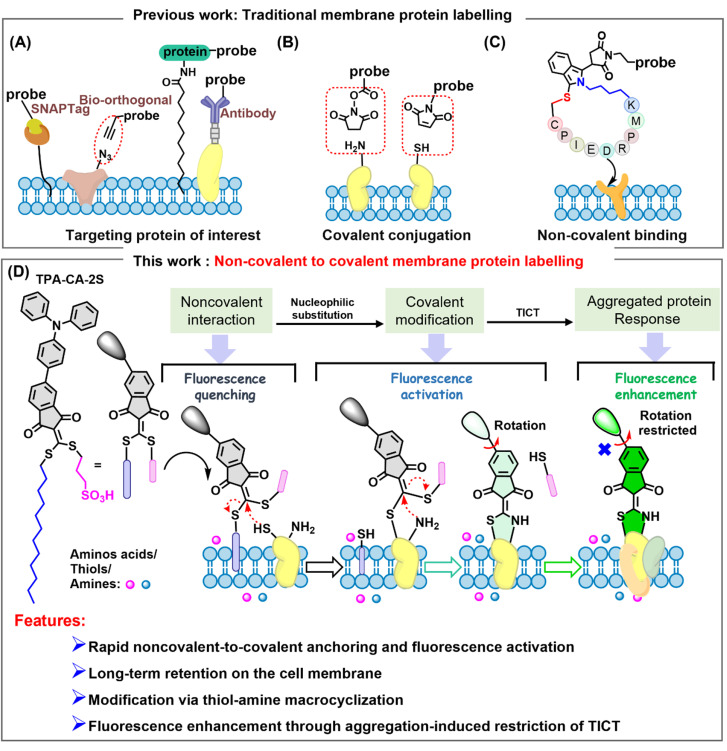
Evolution of strategies for cellular membrane protein labeling. Previous work: (A) genetically encoded fluorescent protein or antibody epitope tagging for targeted protein visualization. (B) Chemically triggered covalent labeling of membrane proteins *via* thiol or amine. (C) Cyclic-peptide based labeling for selective modification of membrane protein receptors. This work: (D) non-covalent to covalent labeling of membrane protein labeling through macrocyclization and real-time monitoring of membrane protein aggregation.

To investigate membrane protein aggregation, researchers have developed a series of biophysical and chemical labeling methods.^9–11^ Foundational biological approaches offer high specificity through genetically encoded systems, such as covalent labeling of a protein of interest with fluorescent dyes *via* HaloTag or SNAP-tag, or through bioorthogonal chemistry, where a reactive handle facilitates a selective “click” reaction with a probe (Fig. 1A).^12–15^ Methods that utilize high-affinity non-covalent bonds to label cell surface proteins, such as antibody-based membrane protein labeling techniques, and approaches relying on hydrophobic interactions for protein tagging have also been developed (Fig. 1A).^16^ Another common strategy relies on nucleophilic substitution reactions with amine or thiol groups present on the cell membrane (Fig. 1B). However, these approaches face high technical thresholds and involve complex procedures, while also posing potential risks due to the disruption of native protein structures.^17–19^ A recently developed non-covalent method utilizing pyrazolone-–protein interactions offers a new modality for probing native protein behavior and enabling long-term staining and facile artificial biorecognition of cell membranes.^20^ Alongside such developments, strategies to improve the specificity and stability of membrane protein labeling have been developed, where fluorescent probes are conjugated to targeting peptides, enabling high-affinity recognition of native membrane protein receptors (Fig. 1C).^21–24^ However, most probes used in this strategy are constitutively fluorescent, and the peptide–cell incubation requires washing steps, which together generate high background signals and hinder the detection of dynamic protein activities such as conformational changes. This limitation underscores the need for new chemical tools that can translate membrane protein dynamics into measurable fluorescence signals.

Inspired by these advances, we developed a non-covalent to covalent strategy for labeling and monitoring cellular membrane proteins through integrating membrane targeting with sensitive aggregation-derived fluorescence responses. Initially, we designed and synthesized an amphiphilic fluorescent probe, named TPA-CA-2S (Fig. 1D), featuring dual functionality that serves as both a membrane-targeting unit and a dual-site reactive centre towards adjacent sulfhydryl from cysteine residues and amine from lysine residues. The action mechanism of TPA-CA-2S involves sequential targeting and labeling (Fig. 1D): (1) amphiphilicity drives initial localization to the lipid bilayer; (2) proximity-induced intramolecular cyclization occurs between protein thiol and amine groups with the release of the moiety; (3) fluorescence enhancement upon protein aggregation by restriction of the twisted intramolecular charge transfer (TICT) effect.^25–28^ This approach provides high specificity in labeling cell membrane proteins through non-covalent targeting to covalent conjugation and reduced non-specific membrane retention, thereby minimizing cytotoxicity. This probe integrates multiple functions together such as targeting, reactivity, and reporting elements. Leveraging the strategy, we achieve chemically stable and selective protein conjugation under physiologically mild conditions. This rational design enables a streamlined and one-pot process, allowing wash-free and long-term labeling of membrane proteins in live cells and subsequent monitoring of their aggregation during chemotherapeutic drug treatments, with visualization achieved through progressively enhanced fluorescence signals.^29^ By integrating “membrane targeting”, “specific labeling” and “aggregation-induced sensing” with a single molecule, this research provides a new strategy for the real-time investigation of membrane protein aggregation dynamics in complex biological contexts.

## Results and discussion

### Reactivity and activation of the probe's fluorescence

**Fig. 2:**
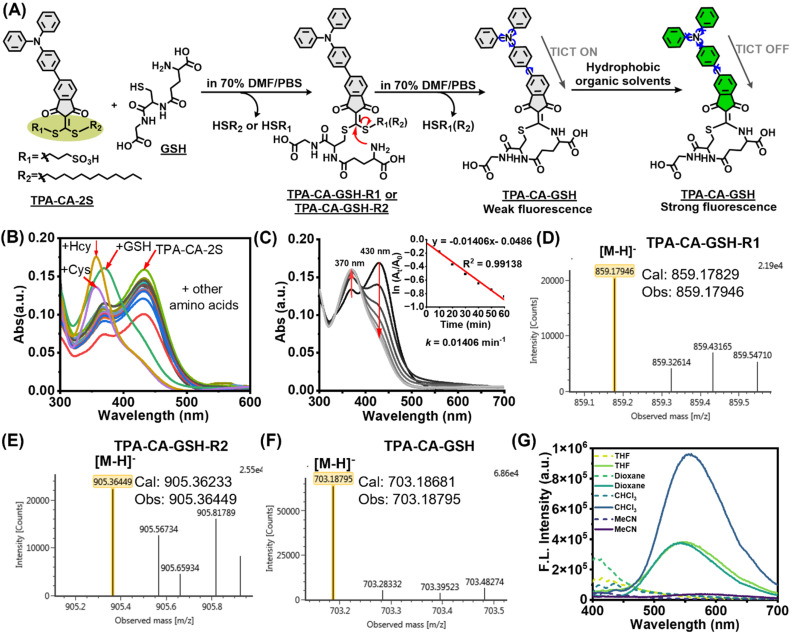
Characterization of the thiol-selective reaction and photophysical properties. (A) Proposed cyclization mechanism of TPA-CA-2S with GSH. (B) UV-vis absorption spectra demonstrating the selectivity of TPA-CA-2S (5 µM) with amino acids (50 µM: Ala, Gly, Thr, Leu, Ser, Arg, Phe, Asp, Met, GSH, Cys, and Hcy) in 70% DMF/PBS buffer (v/v, pH 7.4) for 180 min. (C) UV-vis kinetic profiles of the reaction between TPA-CA-2S (5 µM) and GSH (50 µM). Insets show first-order rate constant calculation in the time range of 0–60 min for each time kinetics at 430 nm. (D)–(F) The products (TPA-CA-GSH-R1, TPA-CA-GSH-R2, and TPA-CA-GSH) of TPA-CA-2S (1 mM) reaction with GSH (5 mM) in 70% DMF/PBS buffer (v/v, pH 7.4) for 60 min. (G) Fluorescence emission spectra of TPA-CA-2S (dashed line) compared with its reaction adduct with GSH (solid line) in solvents of varying polarity (THF, dioxane, CHCl_3_, and MeCN). All experiments were performed at 37 °C.

Initially, the bifunctional probe TPA-CA-2S was synthesized by attaching membrane-anchoring groups (dodecanethiol and sodium 3-mercaptopropylsulfonate) to TPA-CA *via* the thiol–thiol substitution reaction.^30–32^ In 70% DMF/PBS buffer (v/v, pH 7.4), TPA-CA-2S would react with cysteine (Cys), homocysteine (Hcy) and glutathione (GSH) while being unreactive towards other amino acids (Fig. 2B). Subsequently, the mechanistic pathways of this series of small molecules and a peptide featuring different distances between the thiol (–SH) and amino (–NH_2_) were further validated (Fig. S1).

In GSH, the thiol and amine groups are separated by a longer chain (γ-glutamyl linkage). We hypothesized that TPA-CA-2S generates a cyclized product after reacting with GSH and exhibits enhanced fluorescence in low-polarity solvents (Fig. 2A). The reaction induced a significant blue shift in the absorption spectrum: the peak at 430 nm decreased, while a new peak emerged at approximately 370 nm. Kinetic studies confirmed that these reactions were rapid, reaching near-quantitative completion within 60 min (Fig. 2C). This study validated the proposed two-step reaction and fluorescence turn-on mechanisms. High-resolution mass spectrometry (HRMS) characterization of the products obtained from the reaction of TPA-CA-2S with GSH revealed the formation of two distinct mono-adducts (TPA-CA-GSH-R1, *m*/*z* = 906.36391; TPA-CA-GSH-R2, *m*/*z* = 859.17829) (Fig. 2D and E) and a final cyclized species (TPA-CA-GSH, *m*/*z* = 703.18681) (Fig. 2F). As follows, the reactivity of TPA-CA-2S towards *N*-acetyl-l-cysteine (NAC, a thiol-only), Cys, Hcy, and peptide P1 was further investigated using UV-vis and HRMS (Fig. S2–S5). The results revealed that these reactions were accompanied by a blue shift from 430 nm to ∼370 nm. The first-order rate constants (*k*) followed the order: Cys (0.0282 min^−1^) > Hcy (0.0152 min^−1^) > GSH (0.0141 min^−1^) > P1 (0.00805 min^−1^) > NAC (0.0067 min^−1^). Furthermore, HRMS analysis confirmed that the reactions between TPA-CA-2S and these small molecules yielded substitution and cyclized products, with the exception of NAC, which produced a bis-substituted thiol product. These identified intermediates and final products provide strong evidence for the proposed mechanism: an initial thiol–Michael addition followed by an intramolecular cyclization.

The fluorescence of TPA-CA-2S and its cyclized adducts was investigated to probe the cyclization-dependent emission. TPA-CA-2S was found to be non-emissive, whereas its cyclized products with GSH, Cys, Hcy, and P1 exhibited strong fluorescence, which was observed only in low-polarity solvents such as dioxane and THF (Fig. 2G and S2–S5). The corresponding molecule NAC did not exhibit fluorescence signals because it reacted only with –SH groups and did not form a cyclized product (Fig. S2). These findings confirm that intramolecular cyclization functions as a solvent-dependent turn-on switch, activated specifically in hydrophobic microenvironments that restrict molecular motion.^29,32^ In essence, for the observed changes in fluorescence, the displacement reaction with amine is the key to the probe's activation, as we previously reported.^30,33,34^ This mechanism is supported by DFT/TD-DFT studies on the flexible –CH_2_CH_2_SR– linked molecules. Therefore, we propose that TPA-CA-2S in this manuscript enables free bond rotation and favors low-oscillator-strength n → π* transitions, both acting as dominant non-radiative decay channels. Consequently, TPA-CA-2S remains essentially non-emissive even in non-polar solvents, and even TICT has been suppressed. Upon coupling the reaction with both thiol and amine, *i.e.*, TPA-CA-2S-GSH, the amine dominated internal charge transfer results in fluorescence enhancement through the energy-radiative decay path during excitation especially in non-polar solvents with restriction of TICT by the low-polarity environment. In polar solvents, although geometric relaxation is blocked by the rigid structure, the stabilized charge-transfer state re-activates an efficient TICT pathway, resulting in fluorescence quenching. Furthermore, the reaction between the probe and thiol/amine on the protein should actually be macrocyclization even though they would be in proximal positions in principle. However, rigidity of the cyclic structure further affects the fluorescence signal which needs additional exploration in future work.

To further verify the TICT optical response mechanism after labeling with the probe TPA-CA-2S, we investigated the influence of solvent effects. We characterized the photophysical properties of the covalent adducts TPA-CA-2S-GSH and TPA-CA-2S-Hcy. Both conjugates exhibited shorter fluorescence lifetimes in highly polar solvent (MeCN: 2.71 ns and 2.41 ns), whereas longer lifetimes were observed in low-polarity solvent (dioxane: 3.32 ns and 3.63 ns) (Fig. S6 and Table S1). Furthermore, fluorescence spectra of the TPA-CA-2S-Hcy adduct recorded in THF/H_2_O mixtures with water fractions (*f*_W_) ranging from 0% to 100% revealed a continuous and dramatic decrease in emission intensity as the water content increased (Fig. S7). These results demonstrate that the conjugated forms exhibit classic TICT characteristics.

### Response to protein binding and aggregation

**Fig. 3:**
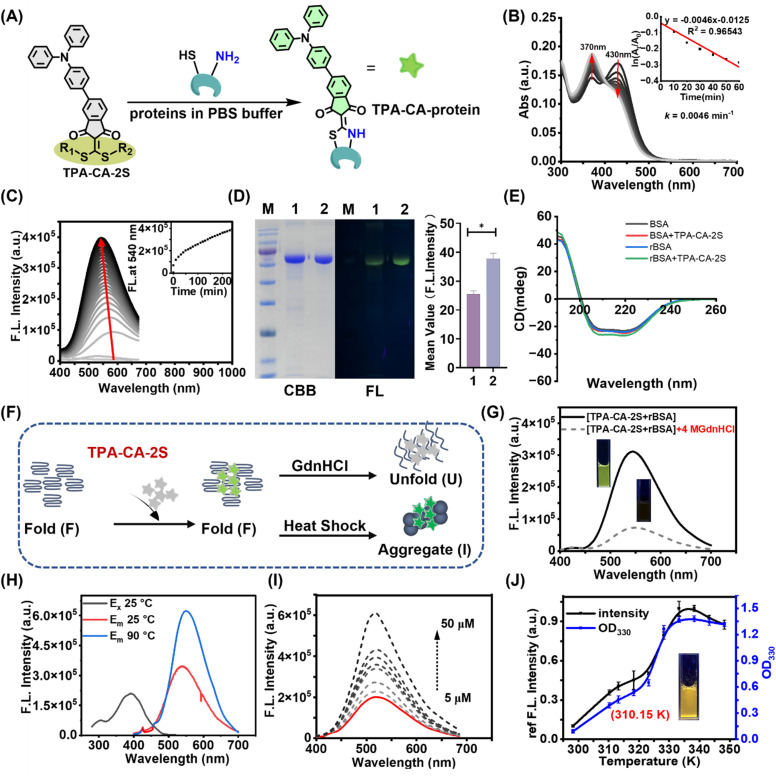
Covalent labeling of proteins and monitoring of protein aggregation. (A) Proposed covalent labeling mechanism of proteins with TPA-CA-2S *via* a reaction with cysteine and lysine residues. (B) and (C) Reaction kinetics between TPA-CA-2S (5 µM) and rBSA (5 µM) monitored by (B) UV-vis absorbance (inset: first-order rate constant calculation in the time range of 0–60 min for each time kinetics at 430 nm) and (C) fluorescence spectra. Inset: fluorescence intensity at 540 nm *versus* time. (D) Left: SDS-PAGE analysis confirming the covalent conjugation of TPA-CA-2S to BSA and rBSA (M: marker; lane 1: TPA-CA-2S + BSA, Lane 2: TPA-CA-2S + rBSA, Coomassie brilliant blue (CBB) stain and fluorescence imaging (FL)); right: quantification of TPA-CA-2S fluorescence intensity of fluorescence imaging, *p < 0.001. (E) CD analysis comparing BSA and rBSA before and after TPA-CA-2S modification. (F) Schematic illustrating the fluorescence response of TPA-CA-2S to different protein conformational states. (G)–(I) Fluorescence spectra of TPA-CA-2S-labeled rBSA: (G) in the presence and absence of GdnHCl, (H) before and after heat denaturation (90 °C, 5 min), and (I) after removal of the free probe by ultrafiltration (3 kDa cutoff). (J) Monitoring thermal aggregation of TPA-CA-2S-r-lysozyme. Temperature-dependent turbidity (optical density at 330 nm: OD_330_) and fluorescence (530 nm). Inset: photograph of the fluorescent solution at 310.15 K.

The confirmation of the TPA-CA-2S fluorescence activation mechanism at the molecular level prompted an investigation into its utility in protein labeling. Specifically, TPA-CA-2S (5 µM) was incubated with native and reduced bovine serum albumin (rBSA), respectively at an equal concentration of 5 µM to assess protein reactivity (Fig. 3A). The reaction with rBSA, which exposes a higher number of free thiols and amino groups, resulted in a significant absorbance change. Specifically, the peak at 430 nm decreased while a new peak emerged at 370 nm. The first-order reaction rate was 0.0046 min^−1^, reaching completion at approximately 60 min (Fig. 3B, inset). These spectral changes, consistent with those observed for small-molecule thiols, indicate effective probe activation. Notably, this reaction also exhibited a gradual, time-dependent fluorescence enhancement, yielding a 61-fold increase in intensity over 180 min (Fig. 3C), directly reflecting progressive covalent binding. In contrast, while native BSA induced a comparable spectral shift in the UV-vis profile (Fig. S8A), with a first-order reaction rate of 0.0017 min^−1^ over approximately 180 min (Fig. S8B), it yielded a substantially lower, 27-fold fluorescence enhancement (Fig. S9). The nearly identical changes observed in the UV-vis spectra suggest that both native and rBSA provide sufficient reactive sites to engage a comparable amount of the TPA-CA-2S probe, likely reaching reaction saturation or equilibrium under the experimental conditions. The dramatic difference in fluorescence enhancement (27-fold for BSA *vs.* 61-fold for rBSA) indicates that the local microenvironment surrounding the covalently bound probe plays a pivotal role (Fig. S9) and this discrepancy can be attributed to the probe's TICT properties. The significantly higher fluorescence signal observed with rBSA, compared to native BSA, is postulated to arise from the more rigid and hydrophobic microenvironment of its partially unfolded conformation, which suppresses the TICT-related non-radiative decay pathway. To validate the covalent binding of TPA-CA-2S to proteins, SDS-PAGE analysis was performed (Fig. 3D, left). The results demonstrated strong fluorescent adduct formation with rBSA (Lane 2) but relatively low labeling of native BSA (Lane 1). Quantitative densitometry of the fluorescent bands revealed that the labeling efficiency of rBSA was higher than that of native BSA (*p < 0.001, *n* = 3 independent gels, Fig. 3D, right), with a significant difference, further confirming the thiol-dependent covalent attachment. Far-UV circular dichroism (CD) spectroscopy on both native and TPA-CA-2S-labeled BSA/rBSA (1 equivalent probe) was performed. As shown in Fig. 3E, the characteristic negative bands at 208 nm and 222 nm of α-helical BSA remain almost unchanged after labeling, and the overall CD spectra of native and labeled proteins are essentially identical. These results demonstrate that our labeling strategy does not perturb protein secondary or tertiary structures.

Based on the hypothesis that the probe's fluorescence is governed by its microenvironment, we predicted that inducing protein unfolding or aggregation would dynamically modulate the signal (Fig. 3F). To test this premise and to further validate the phenomenon of aggregation-induced fluorescence, TPA-CA-2S-BSA/rBSA were employed as an additional model system. Treatment with guanidine hydrochloride (GdnHCl), a chemical denaturant, led to a marked fluorescence decrease (approx. 4-fold for rBSA and 3-fold for BSA; Fig. 3G and S10). This quenching upon unfolding is consistent with increased probe exposure to a more polar aqueous environment, leading to non-radiative relaxation. Additionally, temperature-dependent fluorescence analysis revealed that TPA-CA-2S-rBSA showed signal activation (fluorescence increase upon heating leading to aggregation) at 338.15 K, earlier than TPA-CA-2S-BSA (343.15 K) (Fig. S11). These fluorescence transitions occurred prior to the onset of macroscopic protein aggregation, as indicated by OD_330_ measurements, signifying the probe's sensitivity to early conformational changes. Further heating to 90 °C induced a ∼2-fold fluorescence increase for TPA-CA-2S-rBSA and a ∼3-fold enhancement for BSA (Fig. 3H and S12), consistent with probe activation upon complete protein aggregation. Ultrafiltration (3000 Da cutoff) demonstrated that fluorescence was retained in the high-molecular-weight fraction of the TPA-CA-2S-rBSA mixture, with a minimal signal in the filtrate (Fig. 3I), suggesting that the probe preferentially associates with aggregated or misfolded proteins. Conversely, native BSA showed weak fluorescence, and its filtrate retained a detectable amount of the probe, indicating fewer specific interactions (Fig. S13 and S14). Therefore, the TICT-based probe exhibits conformation-dependent fluorescence behavior, with quenching upon partial unfolding due to increased polarity and marked fluorescence enhancement in the aggregated state owing to restricted intramolecular motion. This highlights its dual sensitivity to both unfolding and aggregation.^35–37^ Furthermore, we labeled recombinant bovine serum albumin (rBSA) with TPA-CA-2S to generate the TPA-CA-2S-rBSA conjugate, which exhibited a fluorescence lifetime of 2.98 ns in its native state. Upon heat-induced aggregation of the protein, the fluorescence lifetime of the conjugate increased to 3.18 ns. In contrast, upon subsequent addition of guanidine hydrochloride (GdnHCl), which disaggregates and denatures the protein, the lifetime decreased to 2.54 ns, accompanied by a substantial reduction in fluorescence intensity (Table S2 and Fig. S15). These controlled *in vitro* experiments demonstrate that TPA-CA-2S is capable of sensing protein aggregation/disaggregation through pronounced changes in emission intensity and lifetime, respectively.

The study was then extended to lysozyme to further explore the probe's aggregation-sensing capabilities. Upon labeling with TPA-CA-2S, protein conformational changes (*e.g.*, unfolding or aggregation) alter the local hydrophobic environment, modulating the TICT process and yielding distinct fluorescence signals. To examine the relationship between protein aggregation and fluorescence, lysozyme, a well-established model with controllable aggregation properties, was selected.^38^ Initial fluorescence monitoring of reduced lysozyme (r-lysozyme, 5 µM) when reacted with TPA-CA-2S (5 µM) showed a gradual, time-dependent increase, reaching a 24-fold enhancement after 2 h (Fig. S16). Comparison of fluorescence-based thermal shift curves with OD_330_ turbidity measurements revealed a positive correlation, demonstrating that the fluorescence intensity increased progressively with protein aggregation (Fig. 3J). Fractionation experiments further confirmed that 92.5% of the fluorescence signal partitioned into the insoluble aggregate fraction, when comparing soluble, folded r-lysozyme with its insoluble aggregated counterpart (Fig. S17).

### Membrane anchoring and protein activated fluorescent labeling

**Fig. 4:**
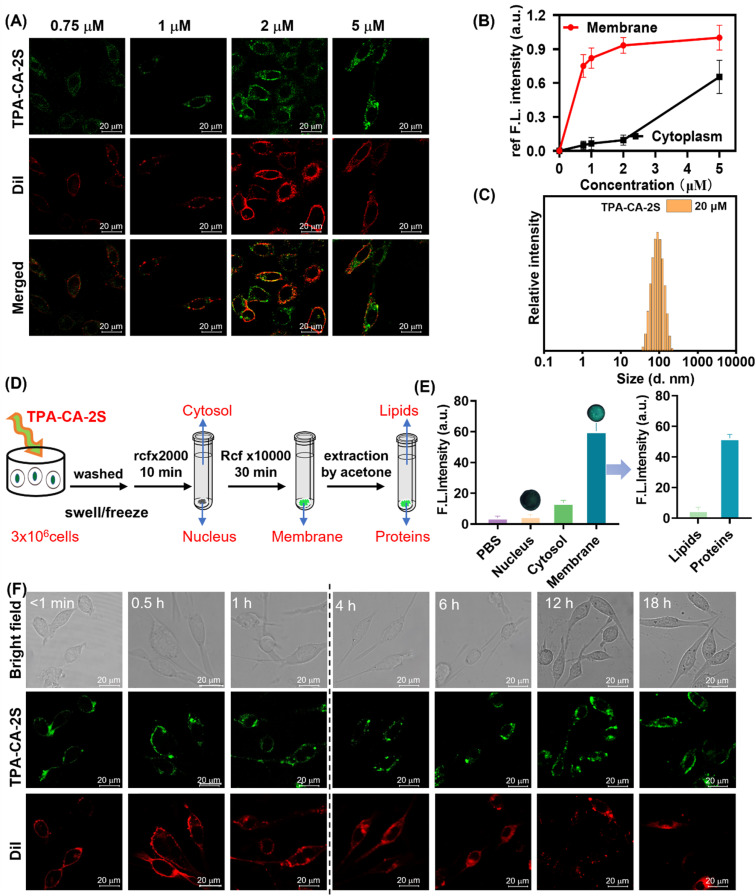
Specific and stable cell membrane labeling using TPA-CA-2S in MC38 cells. (A) Representative colocalization confocal images of MC38 cells stained with Dil (5 µM, 30 min) and subsequently TPA-CA-2S (0.75–5 µM, 40 min). (B) Quantitative analysis of TPA-CA-2S fluorescence intensity in the membrane (M) *versus* intracellular (In) localization as a function of concentration. (C) Size distribution of TPA-CA-2S nanoparticles (20 µM in water) measured by DLS. (D) Schematic of the membrane fractionation protocol. (E) Fluorescence quantification of isolated cellular fractions, confirming the enrichment of TPA-CA-2S in the membrane component (inset: fluorescence images of separated cell membrane components upon irradiation at 370 nm). (F) Colocalization confocal images of Dil (5 µM, 30 min) and TPA-CA-2S (2 µM, 40 min) in MC38 cells at different time points, demonstrating superior long-term membrane retention of TPA-CA-2S compared to Dil. Dil (*λ*_ex_ = 549 nm; *λ*_em_ = 570–620 nm, 30 min), TPA-CA-2S (*λ*_ex_ = 405 nm; *λ*_em_ = 500–560 nm, 40 min), all scale bars = 20 µm.

The cellular biocompatibility and membrane-targeting potential of TPA-CA-2S were further evaluated. CCK-8 cytotoxicity assays demonstrated biocompatibility, with >90% cell viability maintained across concentrations ranging from 0 to 50 µM (Fig. S18), supporting its suitability for biological applications. To assess the probe's targeting ability, concentration-dependent imaging and co-localization analysis with the commercial membrane dye Dil were performed. TPA-CA-2S visualized cell membranes at a concentration as low as 1 µM (Fig. 4A and B), lower than that typically required for phospholipid-inserting dyes of 1–25 µM. However, a higher concentration of 5 µM resulted in the saturation of membrane binding sites, which in turn caused probe internalization. In addition, the difference of staining patterns between the probe and DiI is observed. This selectivity is reflected in the different staining patterns compared to DiI (Fig. 4A): DiI uniformly stains the entire lipid bilayer, yielding continuous membrane fluorescence, whereas TPA-CA-2S exhibits a punctate and heterogeneous pattern across all cell types tested. The staining pattern therefore reflects the native distribution and organization of these target proteins within the membrane, confirming the probe's protein-specific labeling capability. Time-course studies showed rapid membrane labeling within 20 min of incubation with MC38 cells, reaching a fluorescence plateau by 30–40 min (Fig. S19). This membrane-targeting capability was further confirmed by three-dimensional imaging (Fig. S20). As the control group, TPA-CA (lacking the targeting group) exhibited diffuse cytoplasmic fluorescence (Fig. S21), in contrast with the well-defined plasma membrane localization observed for TPA-CA-2S. To delineate the precise membrane interactions of TPA-CA-2S, we extracted cellular components from stained MC38 cells (Fig. 4D). Subsequent fractionation revealed that TPA-CA-2S predominantly associates with membrane proteins rather than phospholipids, as the fluorescence signal was almost entirely detected in the membrane proteins, but not in the nucleus, cytosol, or phospholipids (Fig. 4E). This also suggests that its membrane localization is driven primarily by covalent modification of membrane proteins, rather than simple insertion into lipid bilayers. Complementary dynamic light scattering (DLS) analysis showed that TPA-CA-2S self-assembles into monodisperse nanoparticles (average hydrodynamic diameter of approximately 100 nm) in aqueous solutions (Fig. 4C). This nanoparticle formation synergistically enhances membrane association by (1) increasing the local concentration of the probe at the membrane interface and (2) facilitating membrane interaction *via* its amphiphilic nanostructure.

Next, the imaging performance and stability of the probe TPA-CA-2S on the cell membrane were investigated. Experiments confirmed no discernible difference in green fluorescence signal distribution between washed and wash-free conditions, with fluorescence exclusively localized to the cell membrane in both settings (Fig. S22). This desirable wash-free property simplifies experimental procedures and minimizes potential membrane disturbance, enabling continuous monitoring of membrane dynamics. The long-term retention capability of TPA-CA-2S on the cell membrane was also evaluated. TPA-CA-2S-labeled cell membranes maintained bright green fluorescence for over 18 h (Fig. 4F). In contrast, the commercial plasma membrane dye Dil, which labels *via* phospholipid insertion, showed significant internalization and intracellular fluorescence within 1 h (Fig. 4F). These findings highlight TPA-CA-2S as a covalently reactive targeting probe with biocompatibility, membrane anchoring, wash-free imaging capability, and long-term retention on the cell membrane. These properties pave the way for investigating the dynamic behavior of membrane-associated proteins.

### Detection of redox-driven membrane protein aggregation

Membrane protein disulfide bonds are highly susceptible to thiol–disulfide exchange under pathological or stress conditions. These redox-mediated changes promote intermolecular misfolding, protein aggregation, and disrupted protein–lipid bilayer interactions, ultimately causing membrane dysfunction.^39,40^ Excessive disulfide bond formation enhances aggregation, whereas their reduction may induce misfolding and scrambled intra- or intermolecular disulfide bonds, further accelerating aggregation.^41–43^

**Fig. 5:**
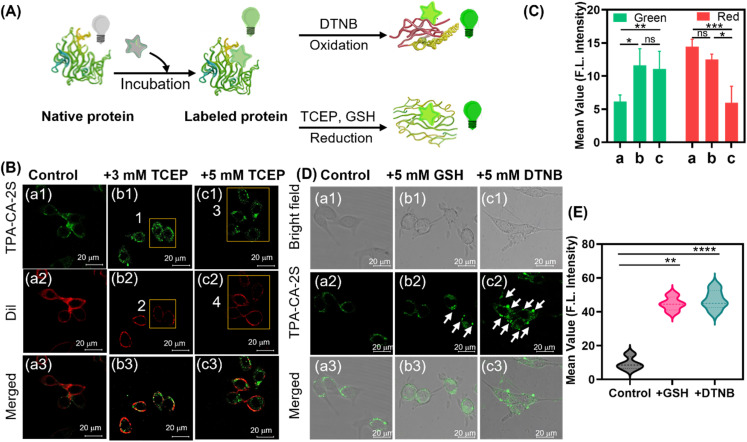
Visualization of redox-driven membrane protein aggregation in live cells. (A) Schematic illustrating the TPA-CA-2S probe detecting protein aggregation on the cell membrane following redox perturbation. (B) Confocal images of MC38 cells pretreated with the reducing agent TCEP (3 or 5 mM, 30 min) and then co-stained with TPA-CA-2S (green) and DiI (red). Yellow boxes indicate TPA-CA-2S aggregation hotspots (1 and 3) and areas of concomitant membrane disruption are indicated by Dil loss (2 and 4). (C) Quantification of TPA-CA-2S fluorescence intensity from (B). (D) Confocal images of MC38 cells showing protein aggregation induced by reducing agents GSH (5 mM) or oxidizing agents DTNB (5 mM) and then stained with TPA-CA-2S. White arrows indicate prominent protein aggregation clusters. (E) Quantification of TPA-CA-2S fluorescence intensity from (D). Dil (*λ*_ex_ = 549 nm; *λ*_em_ = 570–620 nm, 30 min) and TPA-CA-2S (*λ*_ex_ = 405 nm; *λ*_em_ = 500–560 nm, 40 min), all data are presented as mean ± SD (*n* = 20 cells). Statistical analysis was performed using one-way ANOVA. ****p < 0.0001, ***p < 0.001, **p < 0.01, *p ≤ 0.05, and ns > 0.05. Scale bar: 20 µm.

To investigate the impact of redox modulation on membrane protein aggregation, we employed three distinct thiol-reactive agents (Fig. 5A): the reducing agents tris(2-carboxyethyl)phosphine (TCEP), GSH, and the oxidizing agent 5,5′-dithiobis(2-nitrobenzoic acid) (DTNB). These compounds enable precise manipulation of disulfide bond states on the cell surface, providing insights into redox-driven membrane protein misfolding and aggregation.^44,45^ First, we explored the effect of reductive stress. Treatment with TCEP (3 or 5 mM), a potent disulfide bond reducing agent, resulted in a significant 1.5- to 2-fold increase in the green fluorescence intensity of TPA-CA-2S (*p* < 0.01). This manifested as a distinct granular pattern on the cell surface, indicative of heightened membrane protein aggregation (Fig. 5B (regions 1 and 3) and Fig. 5C). Concurrently, the red fluorescence from the membrane dye Dil showed localized signal loss, with an approximate 3-fold decrease in intensity in corresponding regions (Fig. 5B, regions 2 and 4). This suggests that TCEP-mediated reduction of disulfide bonds also compromised membrane integrity, contributing to membrane architecture disruption and promoting protein aggregation by destabilizing protein conformations.

The effects of oxidative stress were subsequently examined. Extracellular treatment with 5 mM GSH, which creates an oxidative environment, induced notable changing of the probe's fluorescence signal. The probe signal changed from a uniform membrane distribution to aggregated states, partially localized in intracellular compartments (Fig. 5D). This was accompanied by a significant 2-fold increase in overall fluorescence intensity (*p* < 0.01) compared to the control group (Fig. 5E). Similarly, treatment with the thiol-oxidizer DTNB (5 mM) caused granular membrane fluorescence and a distinct 3-fold intensity rise (*p* < 0.0001) (Fig. 5D and E). These findings establish that the native state of membrane proteins is sensitive to redox perturbations. Both reduction and oxidation of disulfide bonds induce aggregation, a process reported by the TPA-CA-2S probe in live cells, which highlights the probe's potential for studying pathological processes linked to redox dysregulation.

### Visualization of chemotherapy-induced membrane protein aggregation

The well-established mechanisms of paclitaxel (PTX) and 5-fluorouracil (5-FU) center on their disruption of microtubule dynamics and nucleic acid synthesis. While these actions trigger a cascade of cellular stress responses, it was reasoned that such profound intracellular events would inevitably propagate to the cell surface, altering the integrity and organization of the plasma membrane. Specifically, the cytoskeleton provides a scaffold that provides anchors and confines membrane proteins; its disruption by PTX could therefore liberate these proteins, enabling their aggregation. Similarly, the metabolic and proteotoxic stress elicited by 5-FU can lead to protein misfolding and clustering.^46–48^

**Fig. 6:**
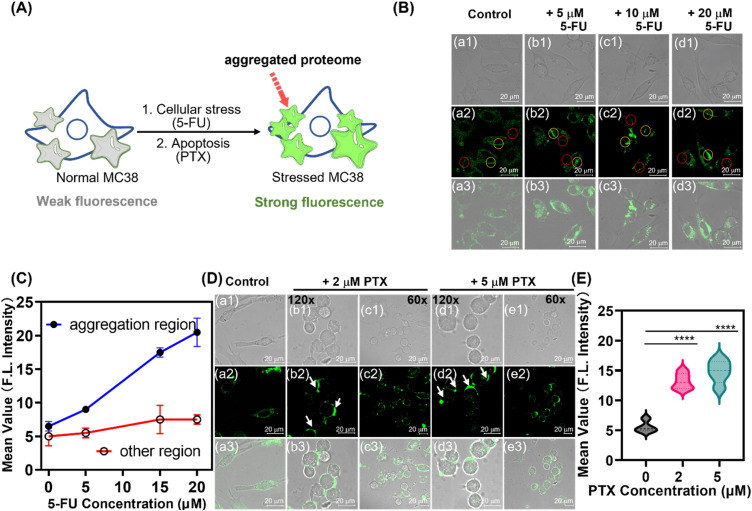
Real-time imaging of membrane protein aggregation induced by chemotherapeutic drugs. (A) Schematic illustrating the TPA-CA-2S fluorescence turn-on mechanism upon binding to aggregated membrane proteins under cellular stress. (B) Confocal microscopy images of MC38 cells treated with varying concentrations of 5-FU for 12 h and then stained with TPA-CA-2S for 40 min. (C) Quantification of mean fluorescence intensity from protein aggregation regions (yellow circles in B) *versus* non-aggregation regions (red circles in B). (D) Confocal images showing membrane protein aggregation (white arrows) in MC38 cells treated with PTX for 12 h and then stained with TPA-CA-2S for 40 min. (E) Corresponding quantification of fluorescence intensity for the paclitaxel treatment groups shown in (D). For all imaging experiments (B and D), cells were stained with 2 µM TPA-CA-2S. Statistical significance was determined using a one-way ANOVA. ****p < 0.0001, ***p < 0.001, **p < 0.01, and *p < 0.05; ns, not significant. Scale bar: 20 µm.

It was therefore postulated that a common consequence of PTX and 5-FU treatment is the induction of membrane protein aggregation and the corresponding fluorescence turn-on response (Fig. 6A). To directly investigate this phenomenon *in situ*, a highly specific and sensitive detection method is required. As such the TPA-CA-2S probe exhibiting negligible fluorescence in its simple covalent binding but dramatic enhancement when bound to protein aggregates was selected. This unique property makes TPA-CA-2S an ideal reporter for monitoring the spatiotemporal dynamics of protein aggregation induced by PTX and 5-FU in cancer cells.

As such the effects of 5-FU, an antimetabolite drug, were investigated. Following a 12 h treatment of MC38 cells with increasing concentrations of 5-FU (5–20 µM), TPA-CA-2S imaging revealed the emergence of distinct, highly fluorescent puncta on the cell membrane, indicative of protein aggregation (Fig. 6B, yellow circles). Quantitative analysis confirmed that the fluorescence intensity within these aggregated regions was approximately 3–4 fold higher than that in adjacent, non-aggregated domains (red circles) and untreated control cells (Fig. 6C). This demonstrates that the TPA-CA-2S's fluorescence enhancement specifically reports localized protein reorganization, rather than a global change in membrane properties.

To determine if this protein aggregation phenomenon was a general response to cytotoxic stress rather than specific to 5-FU, we incubated the cells with PTX, a microtubule-stabilizing agent known to disrupt the cytoskeleton and induce apoptosis. Indeed, a 12 h treatment with PTX (2 or 5 µM) also induced a punctate fluorescence pattern characteristic of membrane protein clustering (Fig. 6D, white arrows). This was accompanied by a comparable 3-/4-fold increase in fluorescence intensity relative to control cells (Fig. 6E). These results demonstrate that TPA-CA-2S functions as a sensitive, real-time reporter of membrane protein aggregation triggered by mechanistically distinct chemotherapeutic agents.

Unlike conventional membrane dyes that primarily signal the loss of membrane integrity, TPA-CA-2S provides molecular-level insights into protein conformational changes and clustering. This establishes it as a suitable tool for studying the cellular response to drug-induced stress.

## Conclusions

Here, we introduce a lipid-anchored fluorescent probe with topology-selective protein labeling capabilities that achieves wash-free, long-term imaging of membrane protein aggregation in living cells by enforcing simultaneous covalent conjugation to a cysteine thiol and a proximate lysine amine on the protein surface. Then the fluorescence signal is enhanced upon stimuli-induced protein aggregation *via* the TICT mechanism. Thus, fluorescence is activated exclusively upon successful dual-site labeling, ensuring that the TICT effect is governed solely by the protein's aggregation state, independent of environmental interference like the hydrophobic milieu of the cell membrane. The probe effectively detects protein aggregation induced by both redox imbalance and chemotherapeutic agents, providing insights into distinct membrane reorganization events. Its biocompatibility, highly selective protein targeting, and wash-free imaging properties underscore its broad utility for long-term live-cell applications. This research provides valuable chemical tools for decoding the intricate structural dynamics of membrane proteins, holding promise for advancing fundamental understanding and targeted therapeutic strategies in cancer and related diseases.

## Author contributions

The conceptualization and design of the study were jointly developed by H. Wei, H. Zhang, and X. Sun; the experimental methodology was established and implemented by H. Wei and H. Zhang; data visualization and figure preparation were collectively completed by H. Wei, L. Xu, K. Wei, W. Bian, Y. Wen, W. Yu, H. Huang, and H. Zhang; funding acquisition and management were handled by H. Zhang and X. Sun; the initial draft of the manuscript was written by H. Wei and edited by L. Xu, K. Wei, W. Bian, Y. Wen, W. Yu, H. Zhang, H. Huang, T. D. James, and X. Sun, participated in the reviewing and editing processes.

## Conflicts of interest

The authors declare that they have no competing interests.

## Supplementary Material

SC-OLF-D5SC07716H-s001

## Data Availability

All data needed to evaluate the conclusions in the paper are present in the paper and/or the supplementary information (SI). Supplementary information: experimental procedures, characterization data (NMR, HRMS), cell imaging, *etc.* See DOI: https://doi.org/10.1039/d5sc07716h.
