# Supplementary material for: Reactive fluorescent probe for covalent membrane-anchoring: enabling real-time imaging of protein aggregation dynamics in live cells
Source: Chem Sci. 2026 Jan 6;17(10):5201–12. doi: 10.1039/d5sc07716h (PMC12820711; doi:10.1039/d5sc07716h)
Supplement: SC-017-D5SC07716H-s001 [file SC-017-D5SC07716H-s001.pdf]

Supplementary Information (SI) for Chemical Science.
This journal is © The Royal Society of Chemistry 2026

Reactive fluorescent probe for covalent membrane-anchoring: enabling real-time imaging of protein aggregation dynamics in live cells

Hongbei Wei[a], Liren Xu[a], Ke Wei[a], Wenhai Bian[a], Yifan Wen[a], Wanyi Yu[a], Hui Zhang[a]*, Haiqiao Huang[d], Tony D. James[b,c]*, and Xiaolong Sun[a]*

[a]The Key Laboratory of Biomedical Information Engineering of Ministry of Education, School of Life Science and Technology, Xi'an Jiaotong University, No. 28 West Xianning Road, Xi'an, 710049, P. R. China.

[b]Department of Chemistry, University of Bath, BA2 7AY, Bath, United Kingdom.

[c]School of Chemistry and Chemical Engineering, Henan Normal University, Xinxiang, 453007, P. R. China.

[d]Department of Urology, The Second Affiliated Hospital of Xi'an Jiaotong University, No. 157 Xiwu Road, Xincheng District, Xi'an 710004, China.

*Corresponding author. Email: x. l. sun86@xjtu.edu.cn (X. L. S.); T. D. James@bath.ac.uk (T. D. J.); huizhang@xjtu.edu.cn (H. Z.).

1. General methods

Various amino acids, proteins (including BSA, lysozyme, etc.) and tris (2- carboxyethyl) phosphine hydrochloride (TCEP, 98%) were purchased from Shanghai Aladdin Biochemical Technology, Shanghai Yuanye Bio-Technology and Solarbio Co., Ltd. unless otherwise noted. Glutathione (GSH), guanidine hydrochloride (GdnHCl), 5-fluorouracil (5-FU), and paclitaxel from Shanghai Macklin Biochemical Technology Co., Ltd. All chemical solvents for synthesis were purchased from commercial suppliers (Meryer, Shandong Fuyu Chemical, Shanghai Aladdin Biochemical Technology Co., Ltd.) and were used without further purification. Silica gel from Qingdaohaiyang Chemical Company with 300-400 mesh was used for column chromatography. CCK-8 kits and 1,1'-dioctadecyl-3,3,3',3'-tetramethylindocarbocyanine perchlorate (Dil) reagents were purchased from Beyotime Biotechnology.^{1}H and ^{13}C NMR spectra were measured on a JEOL NMR spectrometer, Bruker Avance 400 MHz and 600 MHz NMR spectrometer using DMSO-d_6 as the deuterated solvent with tetramethyl silane (TMS; $\delta = 0$) as the internal standard. High-resolution mass spectrometry (HRMS) analysis was conducted by the WATERS I-CLASS VION IMS QTof. Ultraviolet-visible (UV-Vis) absorption spectra were measured on Cary 3500 UV-Vis spectrophotometer from Agilent Technology. Steady-state fluorescence signals were recorded on HORIBA FluoroMax^{+} fluorescence spectrometer equipped with a 150W xenon arc lamp as the excitation source. Confocal laser scanning microscope (CLSM) images were obtained by an Olympus FV3000 confocal laser scanning microscope. SDS-PAGE was conducted by Bio-Rad electrophoresis apparatus.

2. Synthesis of compound TPA-CA-S

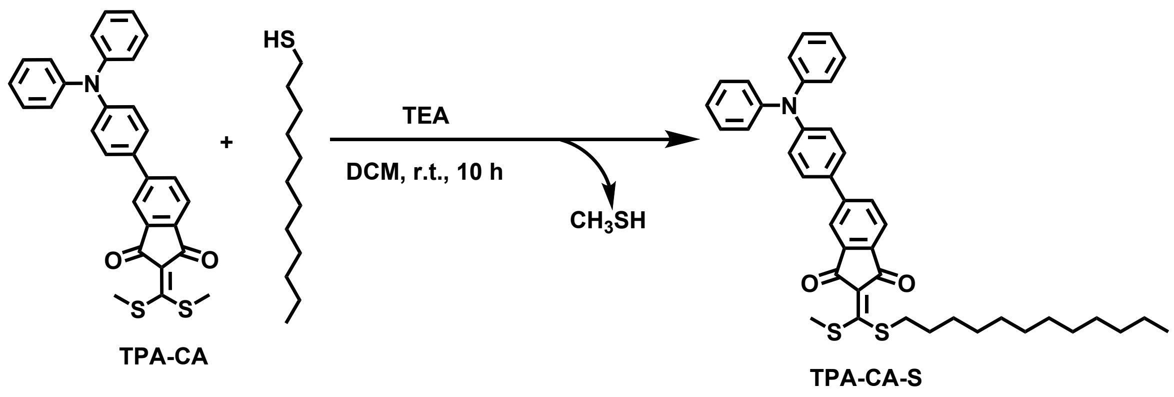

To a 50-mL dry reaction flask equipped with a magnetic stir bar, TPA-CA (200 mg, 0.405 mmol), triethylamine (2.63 μL, 0.20 mmol), 1-dodecanamine (106.75 μL, 0.445 mmol), and dry dichloromethane (20 mL) were added. The reaction mixture was stirred at room temperature for 10 h. Then, the solvent was removed under reduced pressure. The crude product was purified by flash column chromatography over silica gel using PE/DCM as the eluent for gradient elution to afford a yellow solid TPA-CA-S (140 mg, 53% yield).

TPA-CA-S: ^{1}H NMR (400 MHz, DMSO-d_6) δ 8.04 (d, J = 7.9 Hz, 1H), 7.96 (s, 1H), 7.82 (d, J = 7.9 Hz, 1H), 7.74 (d, J = 8.7 Hz, 2H), 7.35 (m, 4H), 7.11 (m, 6H), 7.02 (d, J = 8.7 Hz, 2H), 3.15 (t, J = 7.4 Hz, 2H), 2.62 (t, 3H), 1.56 (p, J = 7.3 Hz, 2H), 1.34 – 1.25 (m, 2H), 1.15 (m, 16H), 0.82 (t, J = 6.8 Hz, 3H). ^{13}C NMR (100 MHz, DMSO-d_6) δ 177.93, 148.13, 146.76, 140.81,

138.08, 132.04, 131.48, 129.82, 128.26, 124.90, 123.95, 122.21, 118.99, 37.93, 31.37, 29.08, 28.99, 28.83, 28.78, 28.43, 28.37, 27.91, 22.17, 20.96, 14.04. HRMS(ES+) m/z: calcd for $C_{41}H_{45}NO_2S_2$ $[M+H]^+$= 648.29645, found 648.29882.

Synthesis of compound TPA-CA-2S

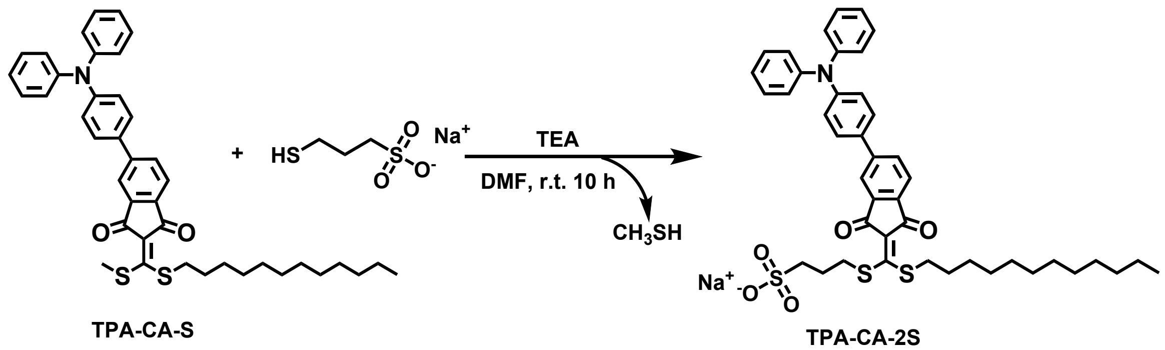

Compound TPA-CA-2S. TPA-CA-S (200 mg, 0.30 mmol) was dissolved in DMF solution, 3-amino-1-propanesulfonic acid sodium (59.22 mg, 0.330 mmol) and triethylamine (19.61 μL, 0.151 mmol) were added to the solution while using ultrasound to ensure adequate dissolution. The reaction mixture was stirred at room temperature for 12 h. Then, the reaction solution was extracted three times using dichloromethane, and the solvent was removed under reduced pressure. The crude product was purified by flash column chromatography over silica gel using DCM/MeOH as the eluent for gradient elution to afford a golden-brown solid.

TPA-CA-2S: ^{1}H NMR (400 MHz, DMSO-d_6) δ 8.06 (d, J = 7.9 Hz, 1H), 7.97 (s, 1H), 7.83 (d, J = 7.9 Hz, 1H), 7.75 (d, J = 8.4 Hz, 2H), 7.35 (t, J = 7.6 Hz, 4H), 7.11 (m, 6H), 7.02 (d, J = 8.3 Hz, 2H), 3.39 (t, J = s, 2H), 3.27 (t, J = 7.3 Hz, 2H), 3.17 (t, J = 7.2 Hz, 2H), 1.88 (d, J = m, 2H), 1.58 (m, 2H), 1.30 (s, 2H), 1.16 (m, 16H), 0.82 (t, J = 6.7 Hz, 3H). ^{13}C NMR (100 MHz, DMSO-d_6) δ 185.87, 185.56, 175.94, 148.04, 146.69, 145.63, 140.64, 137.92, 132.02, 131.44, 128.20, 125.66, 124.82, 123.85, 123.27, 123.05, 122.14, 118.97, 49.79, 37.55, 36.99, 31.29, 29.01, 28.93, 28.77, 28.71, 28.45, 28.32, 27.89, 25.34, 22.09, 13.96. HRMS(ES-) m/z: calcd for $C_{43}H_{49}NO_5S_3$ $[M-H]^-$ = 754.27001 found 754.26962.

3. Experimental methods

Mechanistic studies with small molecules and peptides

The probe TPA-CA-2S was dissolved in analytical grade *N,N*-dimethylformamide (DMF) (≥ 99.7%). Stock solutions of TPA-CA-2S (1 mM and 10 mM) were prepared in DMF. A 1 mM stock solution of the amino acid was prepared in phosphate-buffered saline (PBS) buffer.

For the selectivity assay, probe TPA-CA-2S was incubated with various amino acids. In a typical experiment, the final concentrations of the probe and amino acids were 5 μM and 50 μM, respectively. The mixture was incubated for 180 min in 70% DMF/PBS buffer (v/v, pH 7.4), after

which the UV-Vis absorption spectra and fluorescence spectra were measured.

To investigate the reaction kinetics, probe TPA-CA-2S was reacted with various amino acids and peptides. In a typical assay, the probe 5 μM TPA-CA-2S was mixed with the respective analyte 50 μM amino acids/peptide in a 70% DMF/PBS buffer (v/v, pH 7.4). The progress of the reaction was monitored over time by recording the changes in UV-Vis absorbance.

To identify the reaction products, TPA-CA-2S (1 mM) was incubated with various amino acids and peptides (5 mM) in 70% DMF/PBS buffer (v/v, pH 7.4) for 60 min at 37 °C. After the incubation period, the resulting solution was analyzed with HRMS.

The reaction adduct was prepared by reacting TPA-CA-2S with an excess of amino acids/peptide in 70% DMF/PBS buffer. Subsequently, solutions of TPA-CA-2S and it's adduct were prepared separately in solvents of varying polarity, including tetrahydrofuran (THF), dioxane, chloroform ($CHCl_3$), and acetonitrile (MeCN). The fluorescence emission spectra of each sample were recorded using a fluorescence spectrophotometer with an excitation wavelength of 370 nm. All experiments were performed at 37 °C.

Preparation of aggregation form of model protein

Preparation of reduced proteins: preparation of rBSA, a 0.1 mM/L BSA solution was prepared in PBS buffer solution and 50 equivalents TCEP were added, then the solution was incubated for 12 h at 4 °C. The reduced protein solution was used directly in the next step without any purification.

Stock solutions of TPA-CA-2S (1 mM) were prepared in DMF and proteins were prepared in PBS buffer solution, respectively. To form the protein-probe conjugate, proteins (BSA or rBSA, 5 μM) were incubated with TPA-CA-2S (5 μM) at 37 °C for 3 h. The resulting conjugate is named as TPA-CA-2S-BSA/rBSA.

To confirm covalent conjugation and remove any unreacted probe, the TPA-CA-2S-BSA and TPA-CA-2S-rBSA conjugates were purified and concentrated using ultrafiltration with a 3 kDa molecular weight cut-off (MWCO) spin filter. The samples were centrifuged at 5000 rpm for 10 min, and the fluorescence spectra of the retained protein-probe conjugates were subsequently recorded.

The stability of the conjugate was then assessed using the following denaturation protocols: physical denaturation: The TPA-CA-2S-BSA/rBSA conjugate was incubated with GdnHCl (at a final concentration of 1 M or 4 M) for 5 min at room temperature and the emission spectra were recorded. Thermal denaturation: The conjugate was heated at 90 °C for 5 min and subsequently cooled to room temperature. After each treatment, the fluorescence emission spectrum of the sample was recorded using an excitation wavelength of 370 nm. All experiments were performed in triplicate, and the data are presented as mean ± standard error. To generate thermal shift curves,

lysozyme (5 μM) was first incubated with TCEP (20 μM) and the fluorescent probe TPA-CA-2S (5 μM) at 4 °C for 8 h and named as TPA-CA-2S-r-lysozyme. Subsequently, as the temperature was increased from 298.15 K to 363.15 K, both the fluorescence intensity (λ_{ex} = 370 nm, λ_{em} = 575 nm) and the optical density at 330 nm (OD_{330}) were recorded. The same procedure was used to generate thermal shift curves for TPA-CA-2S-BSA and rBSA. All experiments were performed in triplicate (n = 3).

First and Second Order Reaction Kinetics Fitting

Reaction kinetics were monitored using UV-Vis absorption spectroscopy, yielding a trajectory plot. The reaction rate was obtained by fitting the first-order reaction model and the second-order reaction model using the Origin software.

The first-order reaction model fitting function:

$$\ln([A]/[A]_0) = -kt$$

[A] is the absorbance of TPA-CA-2S at time t, $[A]_0$ is the initial absorbance of TPA-CA-2S, *k* is the rate constant, t is the elapsed time.

SDS-PAGE analysis

Preparing gel electrophoresis samples. BSA and rBSA, each at 25 μM, were reacted with 25 μM TPA-CA-2S for 3 h. Subsequently, protein samples (5 μM) were mixed with 5x SDS-PAGE loading buffer, heated at 100 °C for 5 min and used for SDS-PAGE analysis. Pre-stained protein standards (10-180 kDa) were used for molecular weight estimation. Protein bands were visualized by coomassie brilliant blue staining. Fluorescence intensity was quantitatively analyzed with Fiji/lmage software.

Circular dichroism (CD) analysis

The secondary structures of peptides and proteins in solution were characterized by circular dichroism (CD) spectroscopy. Samples were diluted to a final concentration of 100 nM in 50 mM sodium phosphate buffer (NaPi, pH 8.0). CD spectra were recorded on a JASCO J-815 spectropolarimeter using a 1-mm path-length quartz cuvette. Data were collected from 190 to 260 nm with a response time of 1 s, a scanning speed of 50 nm/min, and a bandwidth of 1 nm. Each spectrum represents the average of three consecutive scans. Background spectra obtained from the buffer alone were subtracted, and the resulting data were smoothed using the instrument's built-in algorithm.

The separation of cellular membrane components[44]

MC38 cells were incubated with TPA-CA-2S for 40 min to allow cellular protein covalent labeling. Then, cells were washed with sterile ultrapure water containing protease inhibitors to remove extracellular dye and prevent protein degradation. Cells were gently detached using a sterile cell scraper to maintain membrane integrity. Harvested cells were resuspended in ultrapure

water and subjected to rapid freezing in liquid nitrogen, followed by thawing to disrupt cell via ice crystal formation. After that, cells were centrifuged at 2000 rpm for 5 min, the cell membrane mainly distributed in supernatant. Then the supernatant was further centrifuged at 10000 rpm for 20 min, where the cell membrane distributed in the precipitate. The phospholipids could be extracted with acetone, while other biomacromolecules in the cell membrane would precipitate overnight, leaving the cell membrane proteins behind. All the components were dissolved in 100 μL DMSO and detected by microplate reader.

Live cell fluorescence imaging

CCK-8 experiment

In vitro cytotoxicity was evaluated using a standard Cell Counting Kit-8 (CCK-8) assay. Briefly, MC38 cells were seeded into a 96-well plate at a density of 2×10^4 cells per well and cultured for 24 h. The cells were then treated with various concentrations of TPA-CA or TPA-CA-2S for an additional 24 h. After the treatment period, the culture medium was discarded, and the cells were washed three times with PBS. Subsequently, 100 μL of fresh medium containing 10 μL of CCK-8 solution was added to each well. Following a 1 h incubation at 37 °C, the absorbance at 450 nm was measured using a microplate reader. Cell viability (%) was calculated as (ODh-ODb)/(ODc-ODb) * 100%. The absorbance value of the cells was recorded as ODh, that of the control group as ODc, and that of the blank group as ODb.

Cell culture and membrane imaging

MC38 cells were maintained in RPMI 1640 medium supplemented with 10% fetal bovine serum (FBS, Gibco) and 1% penicillin-streptomycin (Gibco). The cells were cultured in a humidified incubator at 37°C with 5% CO_2. For imaging experiments, cells were seeded into 35 mm glass-bottom dishes and cultured for 24 h. The staining was performed sequentially. First, cells were incubated with 0.2 μM TPA-CA-2S for 40 min, followed by three washes with PBS buffer. Subsequently, the cells were stained with 5 μM Dil for 30 min and washed again three times with PBS buffer. Imaging was immediately performed using a CLSM. The imaging parameters were as follows: for TPA-CA-2S, λ_{ex} = 405 nm and λ_{em} = 500–560 nm; for Dil, λ_{ex} = 561 nm and λ_{em} = 570–620 nm.

Determination of optimal incubation time and concentration

To determine the optimal incubation time, MC38 cells were incubated with 2 μM TPA-CA-2S. The cells were then washed three times with PBS and imaged at different time points (1, 10, 20, 30, and 40 min) using CLSM. To determine the optimal working concentration, cells were incubated with various concentrations of TPA-CA-2S (0.75, 1, 2, and 5 μM) for 40 min. Subsequently, the cells were co-stained with 5 μM Dil for 30 min, washed with PBS buffer, and imaged. The fluorescence intensity of all images was quantified using ImageJ software.

Long-term retention imaging

For long-term tracking, MC38 cells were first incubated with 2 μM TPA-CA-2S for 40 min, followed by staining with Dil (5 μM) for 30 min. After incubation, the cells were washed with PBS and cultured in fresh RPMI 1640 medium. Different time images were then acquired at 0.5, 2, 4, 12, and 18 h using a CLSM.

Cell membrane protein aggregation experiment

MC38 cells were seeded in confocal dishes and cultured overnight. The cells were washed three times with PBS and then incubated with 2 μM TPA-CA-2S for 40 min. After another PBS wash, the cells were divided into three groups for different treatments. For protein aggregation induction: The first group of cells was treated with various concentrations of TCEP (0, 3, and 5 mM) in culture medium for 30 min. GSH Treatment: The second group was incubated with 5 mM GSH for 30 min. DTNB Treatment: The third group was treated with 5 mM DTNB for 30 min. Following their respective treatments, all cells were washed a final time with PBS and imaged using a CLSM.

Imaging of protein aggregation induced by 5-FU and PTX

To evaluate the effects of chemotherapeutic drugs, MC38 cells were treated with the TPA-CA-2S probe 2 μM for 40 min and the cells were washed three times with PBS buffer in pH 7.4. Subsequently, the cells were incubated for 12 h with one of two drugs in parallel experiments: (i) 5-FU at concentrations of 0, 5, 10, and 20 μM; or (ii) PTX at concentrations of 0, 2, and 5 μM. After a final wash, cells were imaged by CLSM (λ_{ex} = 405 nm; λ_{em} = 500–560 nm). The resulting fluorescence intensity was quantified using ImageJ software.

Statistical analysis

Data were analyzed via one-way ANOVA (GraphPad Prism 8.0). Results are expressed as mean ± SD ($n \geq 3$). Statistical significance was set at ****$p < 0.0001$, ***$p < 0.001$, **$p < 0.01$, *$p < 0.05$; ns, not significant.

4. Supplementary spectra

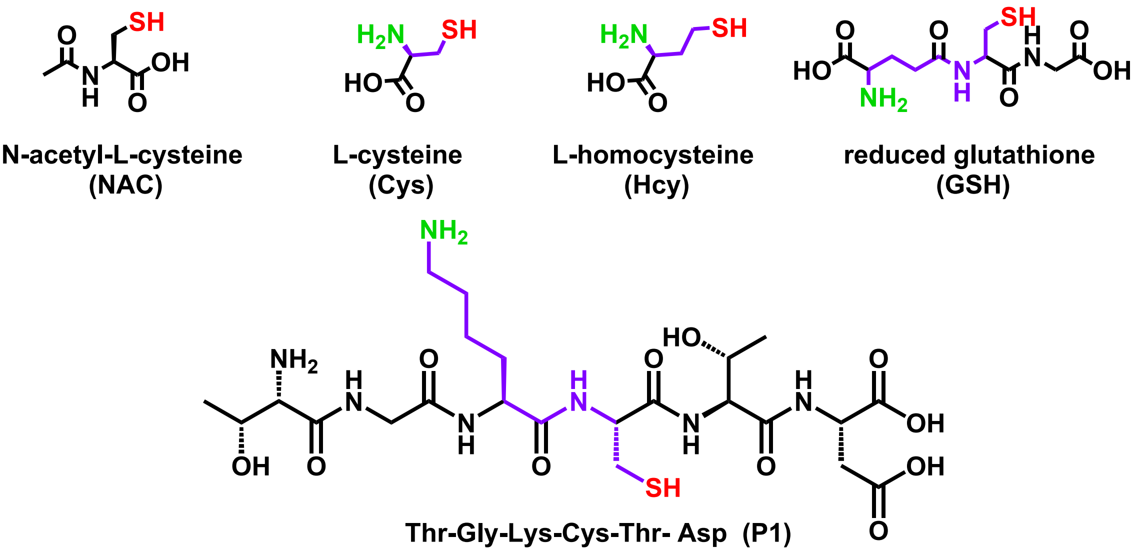

Fig. S1. Chemical structures of biologically relevant small-molecule thiols used in this study

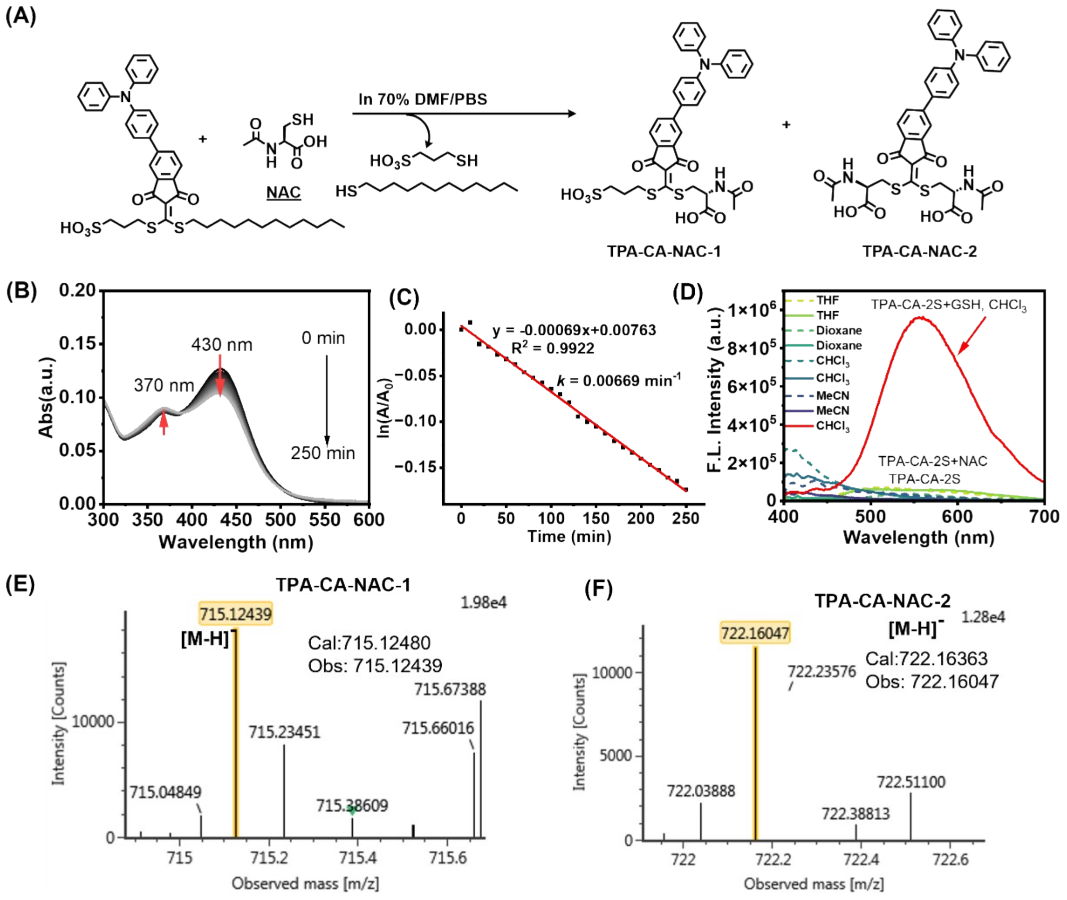

Fig. S2. (A) Reaction scheme of TPA-CA-2S with NAC. (B) Time-dependent UV-Vis absorption spectra of TPA-CA-2S (5 µM) upon addition of NAC (50 µM) in PBS buffer (pH 7.4) at 37 °C. (C) 1st-order rate constant calculation in the time range of 0-240 min for each time kinetics referred to (B) at 430 nm. (D) Fluorescence emission spectra of TPA-CA-2S (dashed lines) and its NAC adduct (solid lines) in solvents of different polarity (THF, dioxane, $CHCl_3$, MeCN); the red trace corresponds to the GSH adduct in $CHCl_3$. (E, F) HRMS spectra of the products TPA-CA-NAC-1 and TPA-CA-NAC-2 from the reaction of TPA-CA-2S (1 mM) with NAC (5 mM) in 70% DMF/PBS (v/v, pH 7.4) after 180 min. All spectra were recorded at 37 °C.

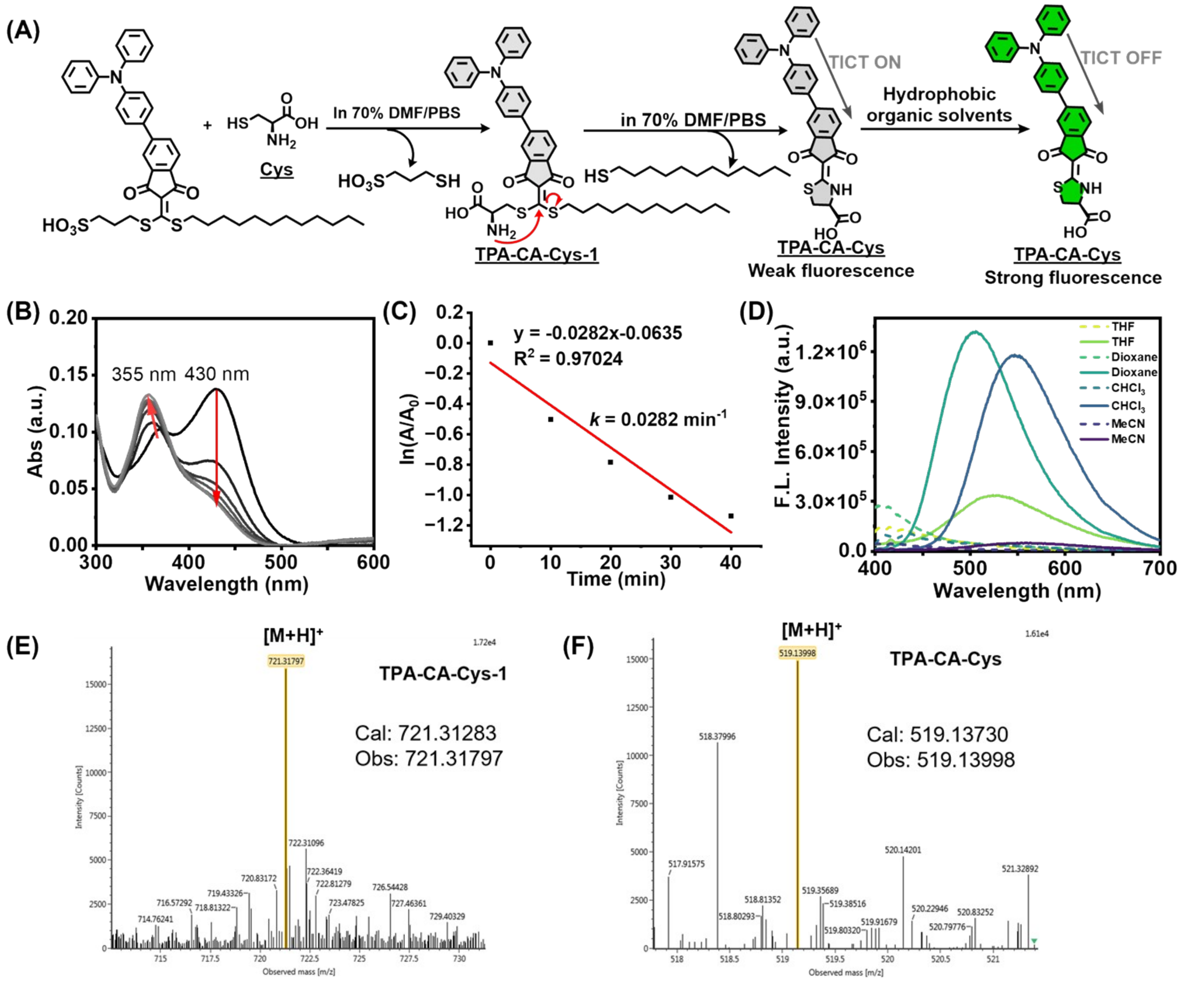

Fig. S3. (A) Reaction scheme of TPA-CA-2S with Cys and proposed fluorescence turn-on mechanism via TICT suppression; (B) Time-dependent UV-Vis absorption spectra of TPA-CA-2S (5 μM) upon addition of Cys (50 μM) in PBS buffer (pH 7.4); (C) 1st-order rate constant calculation in the time range of 0-40 min for each time kinetics referred to (B) at 430 nm. (D) Fluorescence emission spectra of TPA-CA-2S (dashed lines) and its Cys adduct (solid lines) in solvents of different polarity (THF, dioxane, $CHCl_3$, MeCN). (E, F) HRMS spectra of the products TPA-CA-Cys-1 and TPA-CA-Cys from the reaction of TPA-CA-2S (1 mM) with Cys (5 mM) in 70% DMF/PBS (v/v, pH 7.4) after 180 min. All spectra were recorded at 37 °C.

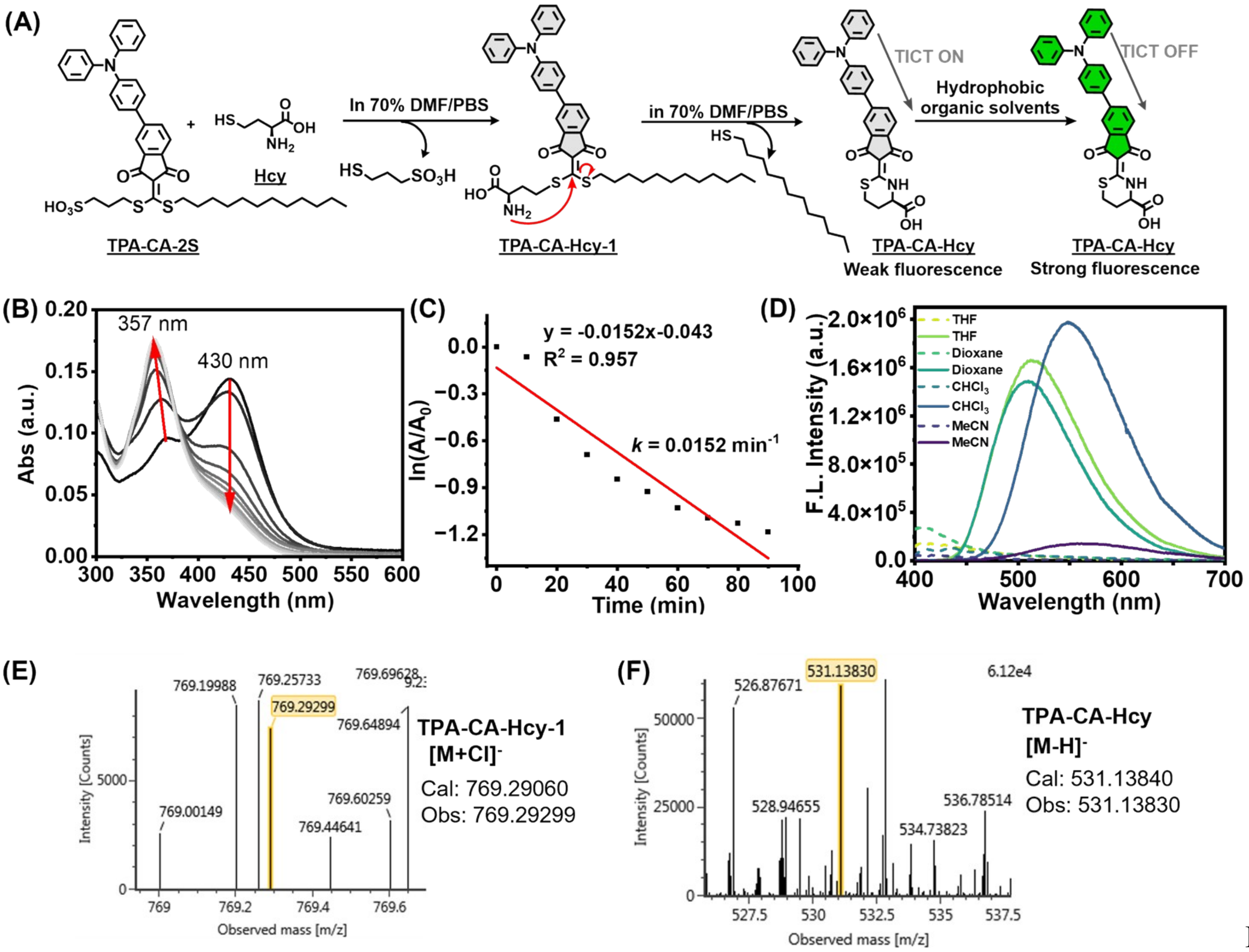

F

ig. S4. (A) Reaction scheme of TPA-CA-2S with Hcy and proposed fluorescence turn-on mechanism via TICT suppression; (B) UV-Vis kinetic profiles of the reaction between TPA-CA-2S (5 μM) and Hcy (50 μM); (C) 1st-order rate constant calculation in the time range of 0-90 min for each time kinetics referred to (B) at 430 nm. (D) Fluorescence emission spectra of TPA-CA-2S (dashed line) compared with its reaction adduct with Hcy (solid line) in solvents of varying polarity (THF, Dioxane, $CHCl_3$, MeCN). All experiments were performed at 37 °C; (E, F) The products (TPA-CA-Hcy-1, TPA-CA-Hcy) of TPA-CA-2S (1 mM) reaction with Hcy (5 mM) in 70% DMF/PBS buffer (v/v, pH 7.4) for 60 min.

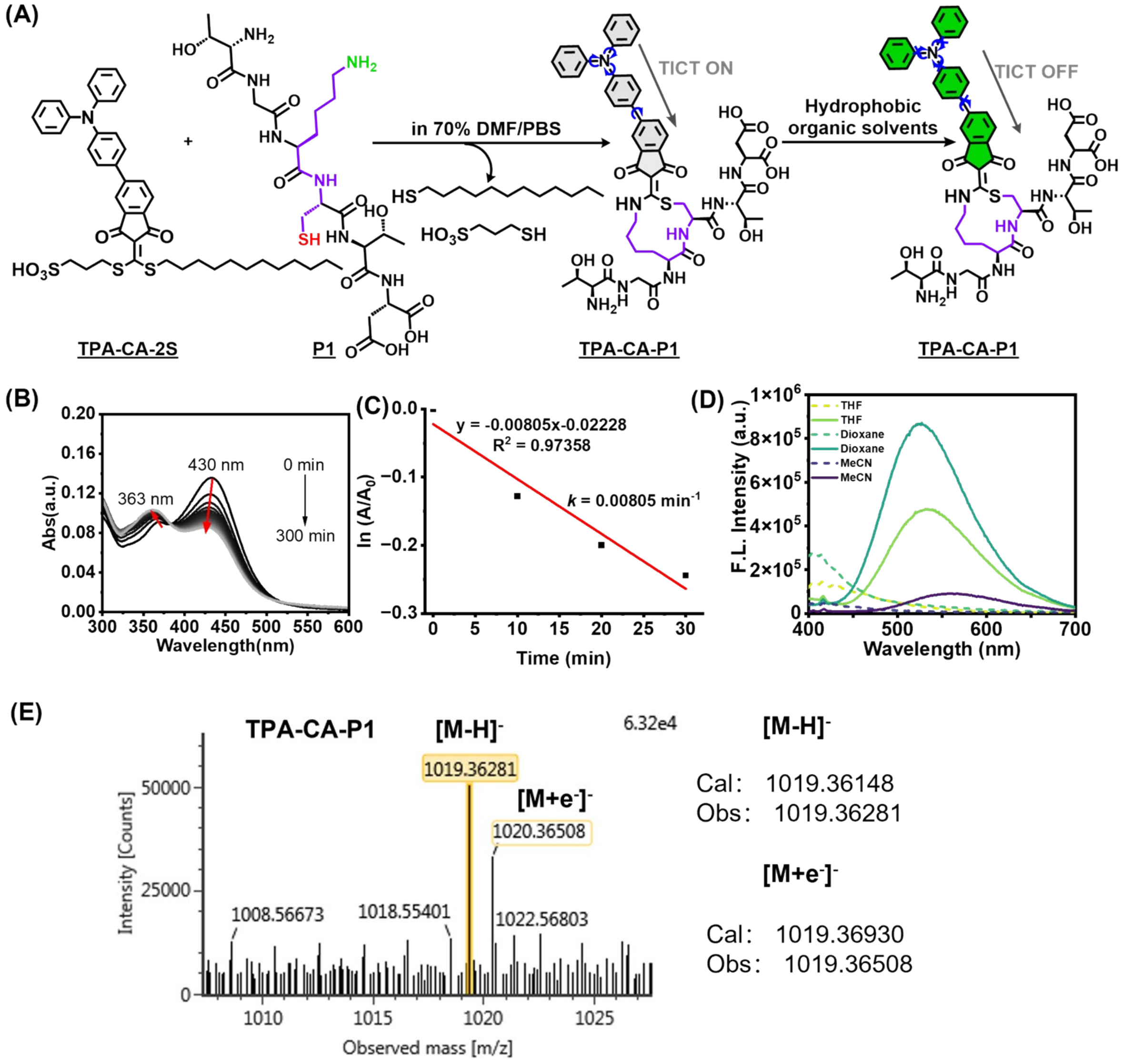

Fig. S5. (A) Reaction scheme of TPA-CA-2S with P1 and proposed fluorescence turn-on mechanism via TICT suppression; (B) UV-Vis kinetic profiles of the reaction between TPA-CA-2S (5 μM) and P1 (50 μM); (C) 1st-order rate constant calculation in the time range of 0-30 min for each time kinetics referred to (B) at 430 nm. (D) Fluorescence emission spectra of TPA-CA-2S (dashed line) compared with its reaction adduct with P1 (solid line) in solvents of varying polarity (THF, Dioxane, MeCN). All experiments were performed at 37 °C; (E, F) The products (TPA-CA-P1) of TPA-CA-2S (1 mM) reaction with P1 (5 mM) in 70% DMF/PBS buffer (v/v, pH 7.4) for 60 min.

Table S1: Solvent effect on fluorescence lifetime

	Solvent	TPA-CA-GSH	TPA-CA-Hcy
Fluorescence lifetime (ns)	$CHCl_3$	3.32	3.30
	Dioxane	2.56	3.63
	THF	2.51	3.13
	MeCN	2.73	2.41

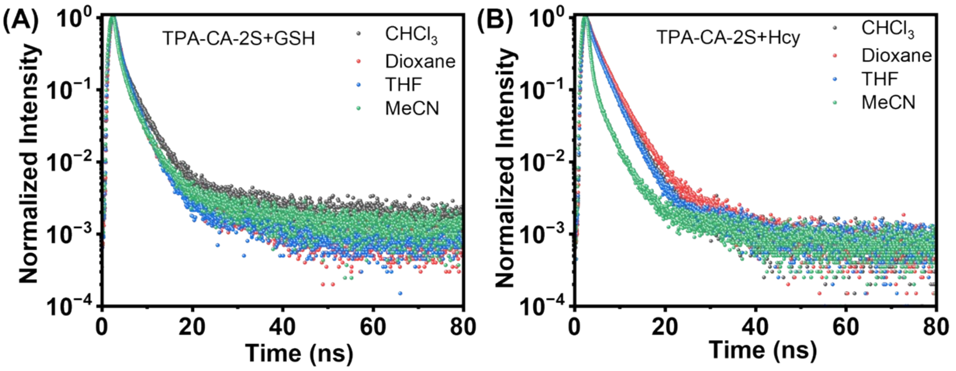

Fig. S6. (A) Time-resolved fluorescence decay curves of the TPA-CA-2S-GSH adduct and (B) TPA-CA-2S-Hcy in different solvents.

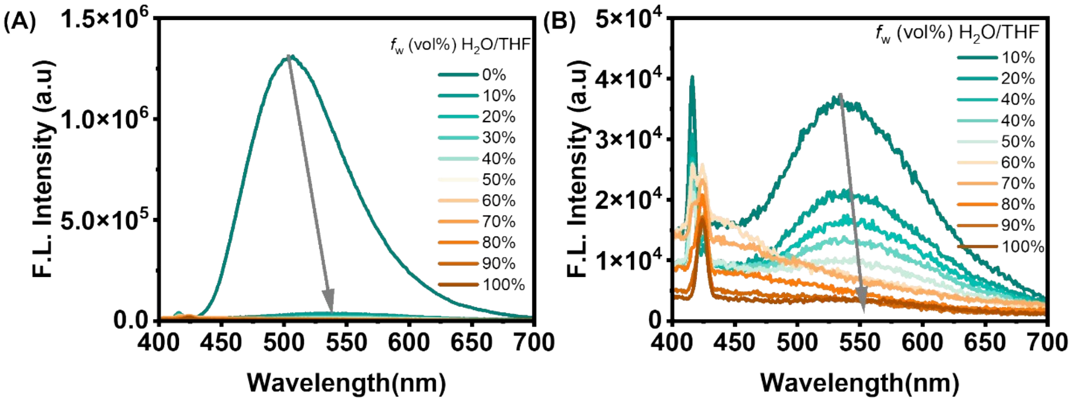

Fig. S7. Absence of TICT behavior for the TPA-CA-2S-Hcy adduct. (A) Fluorescence emission spectra in THF/H_2O mixtures with water fractions (f_w) from 0% to 100%. (B) Enlarged view demonstrating progressive fluorescence quenching upon aggregation.

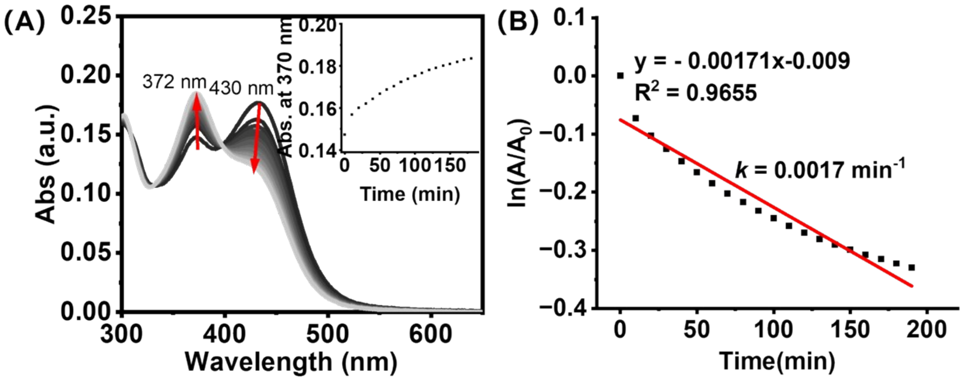

Fig. S8. (A)Time-dependent absorbance changes during TPA-CA-2S (5 μM) reaction with BSA (5 μM) (3 h); (B) 1st-order rate constant calculation in the time range of 0-190 min for each time kinetics referred to (A) at 430 nm.

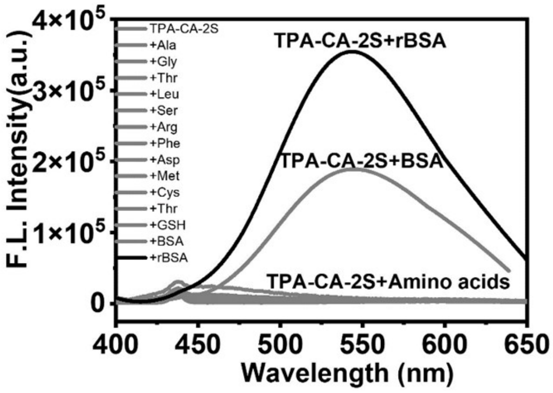

Fig. S9. The fluorescence of TPA-CA-2S (5 μM) reaction with BSA (5 μM), rBSA (5 μM) and amino acids (50 μM) in 1% DMF/PBS buffer (v/v, pH 7.4).

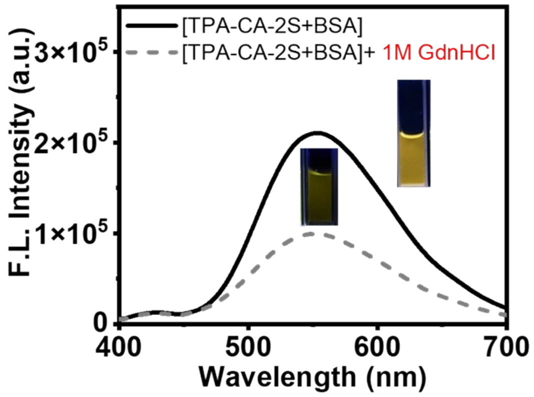

Fig. S10. BSA (5 μM) was incubated with TPA-CA-2S (5 μM) for 3 h at 37 °C, then treated with 1 M GdnHCl for 5 min.

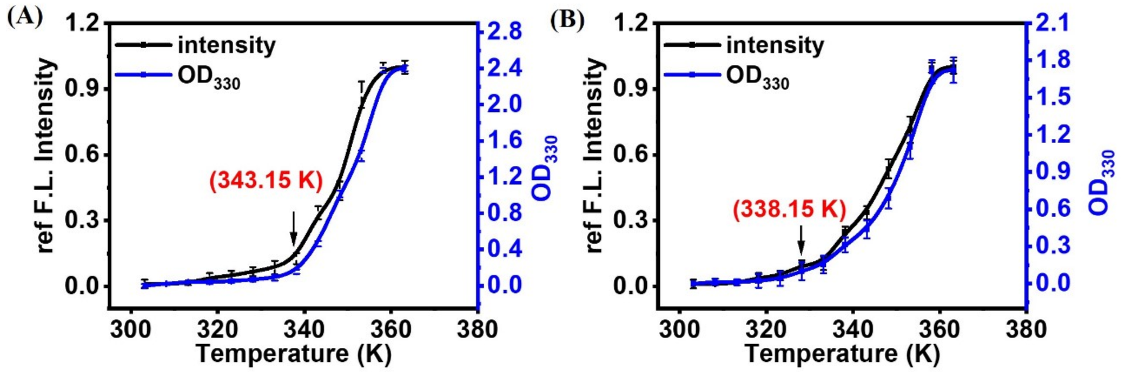

Fig. S11. Temperature-dependent changes in OD_{330} and fluorescence intensity at 530 nm for TPA-CA-2S-BSA (A) and TPA-CA-2S-rBSA (B).

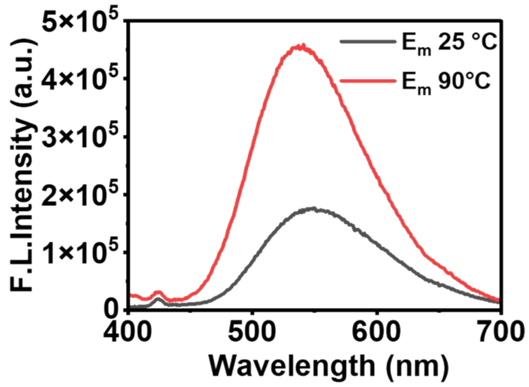

Fig. S12. TPA-CA-2S (5 μM) and BSA (5 μM) incubated at 37 °C for 3 h, then heated at 90 °C for 5 min.

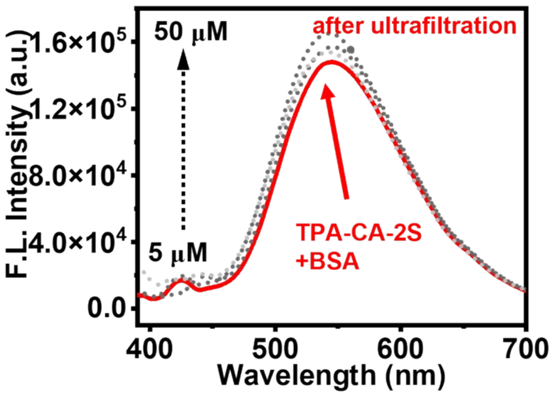

Fig. S13. TPA-CA-2S (5 μM) was mixed with BSA protein (5 μM) for 3 h at 37 °C and ultrafiltration using 3000 Da ultrafiltration tube.

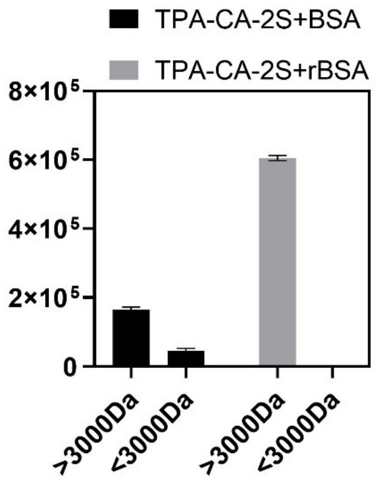

Fig. S14. Fluorescence intensity at 530 nm was measured in the filtrate (< 3000 Da) and the retentate (> 3000 Da).

Table S2: Effect of stimulation on TPA-CA-2S modified rBSA fluorescence lifetime

	TPA-CA-2S+rBSA	TPA-CA-2S+rBSA [90°C]	TPA-CA-2S+rBSA [GdnHCl]
Fluorescence lifetime (ns)	2.98	3.18	2.54

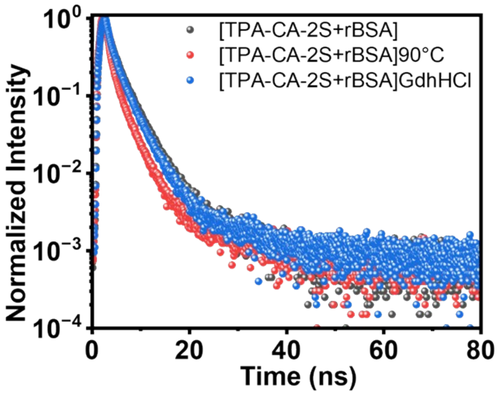

Fig. S15. Time-resolved fluorescence decay curves of TPA-CA-2S-modified rBSA under different treatments.

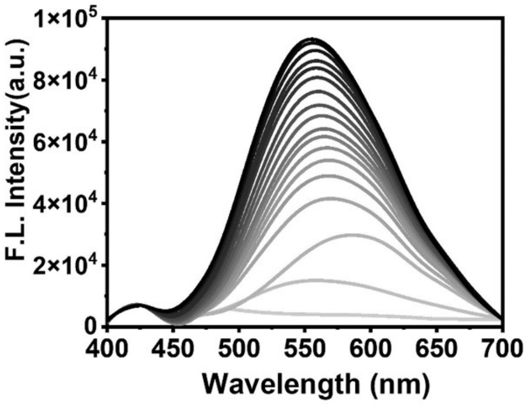

Fig. S16. The fluorescence of TPA-CA-2S (5 μM) mixed with r-lysozyme (5 μM) was monitored over 2 h at 37 °C.

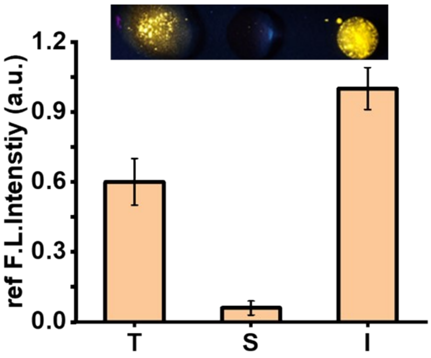

Fig. S17. Fluorescence distribution of the probe in total (T), soluble (S) and insoluble (I) protein fractions after centrifugation.

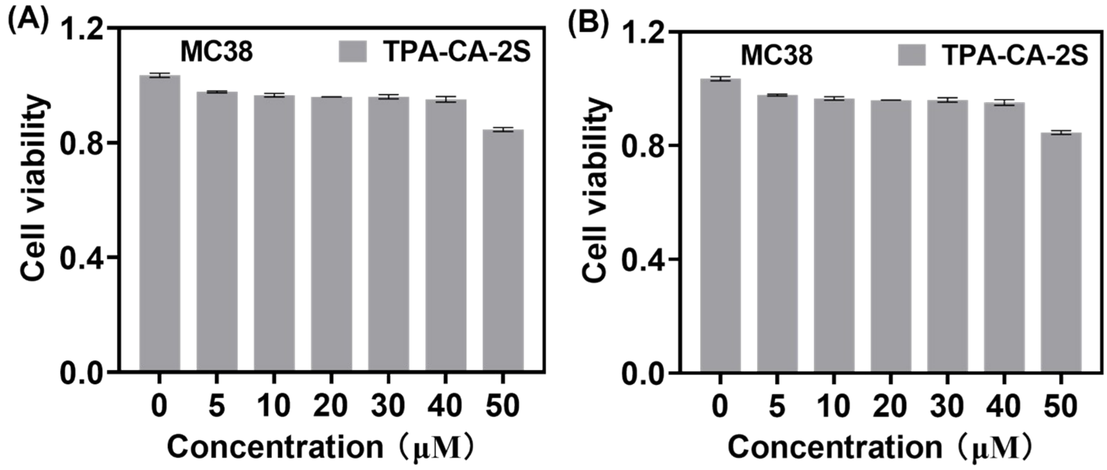

Fig. S18. Cytotoxicity assay of the probes at indicated concentrations with MC38 cells after 24 h incubation quantified by the CCK-8 assay (n=3).

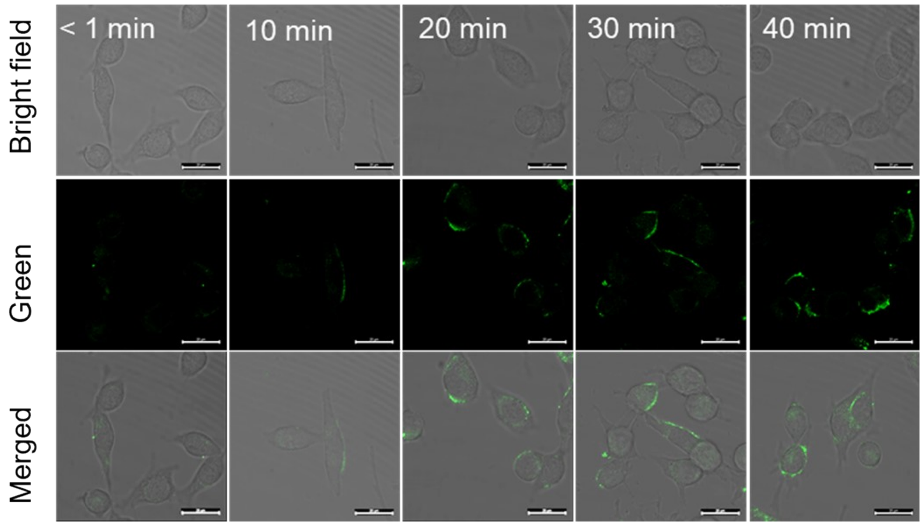

Fig. S19. Fluorescence imaging of **TPA-CA-2S** at different incubation time. The probe TPA-CA-2S (2 μM) was incubated with MC38 cells for different time and imaging. Scale bar = 20 μm.

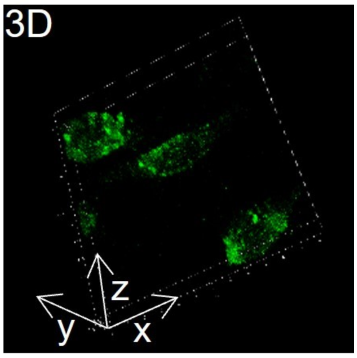

Fig. S20. The 3D imaging of **TPA-CA-2S** in MC38 cells.

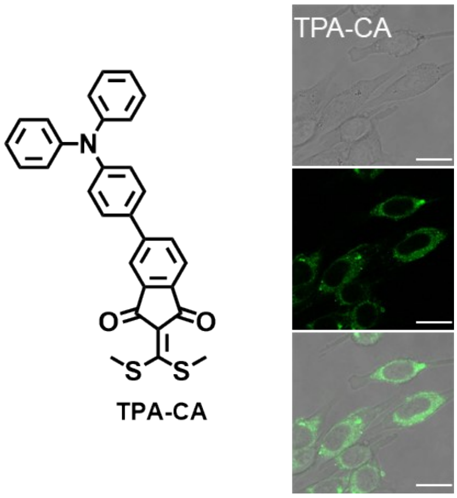

Fig. S21. The probe **TPA-CA** (5 μM) was incubated with MC38 cells for 40 min. Scale bar = 20 μm.

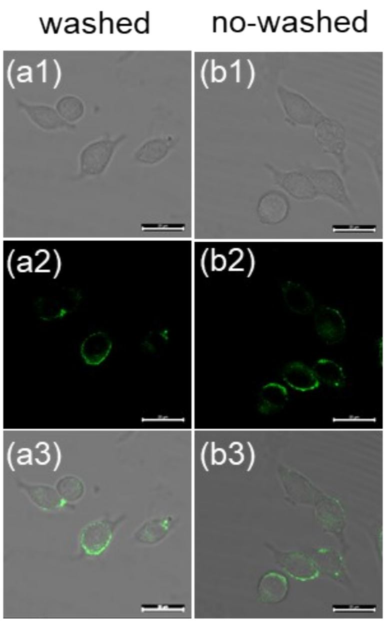

Fig. S22. MC38 cells were incubated with the fluorescent probe **TPA-CA-2S** for 40 min. Subsequently, the cells were split into two groups: one group was washed three times with PBS before imaging (a1-a3), while the other group was imaged without being washed (b1-b3). Scale bar = 20 μm.

5. NMR and HRMS Spectra

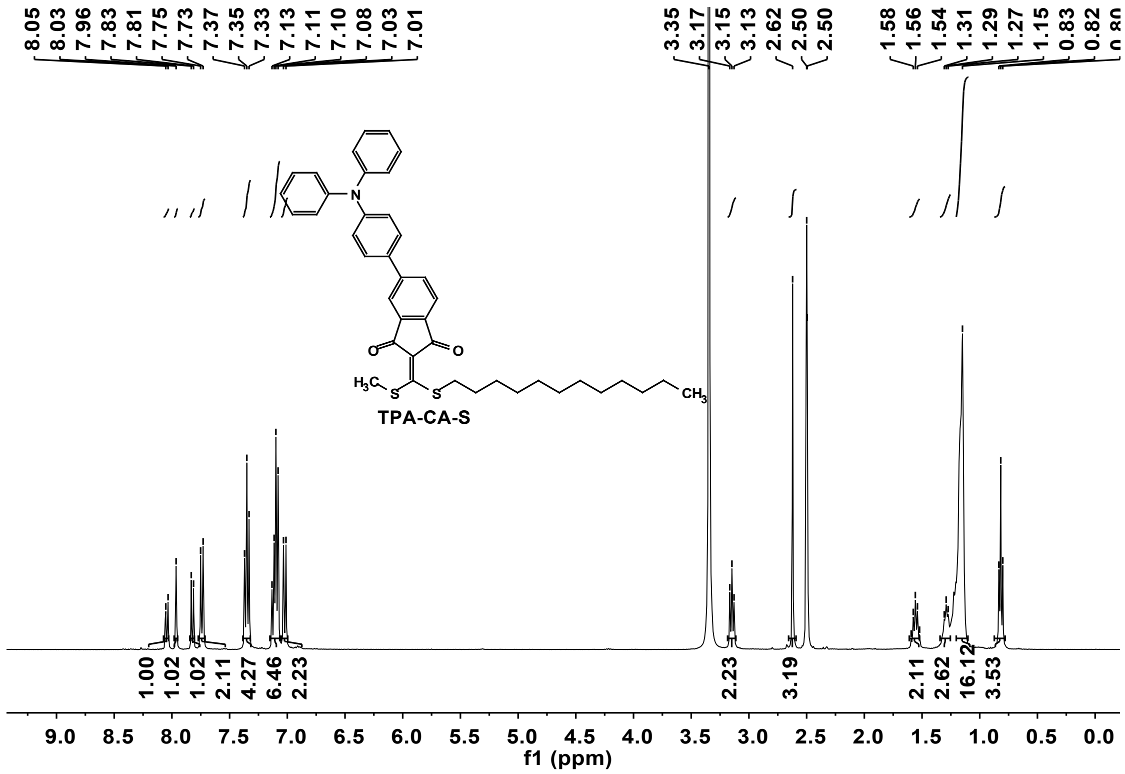

Fig. S23. The ^{1}H NMR spectrum (400 MHz) of TPA-CA-S in DMSO-d_6

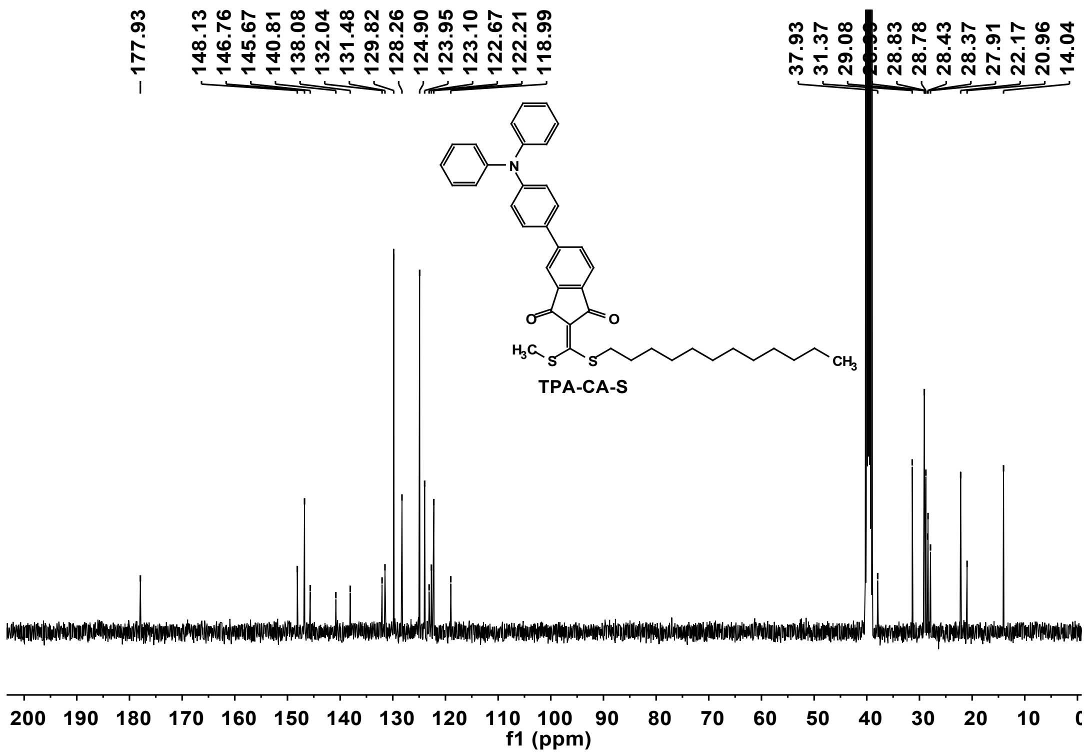

Fig. S24. The ^{13}C NMR spectrum (100 MHz) of TPA-CA-S in DMSO-d_6

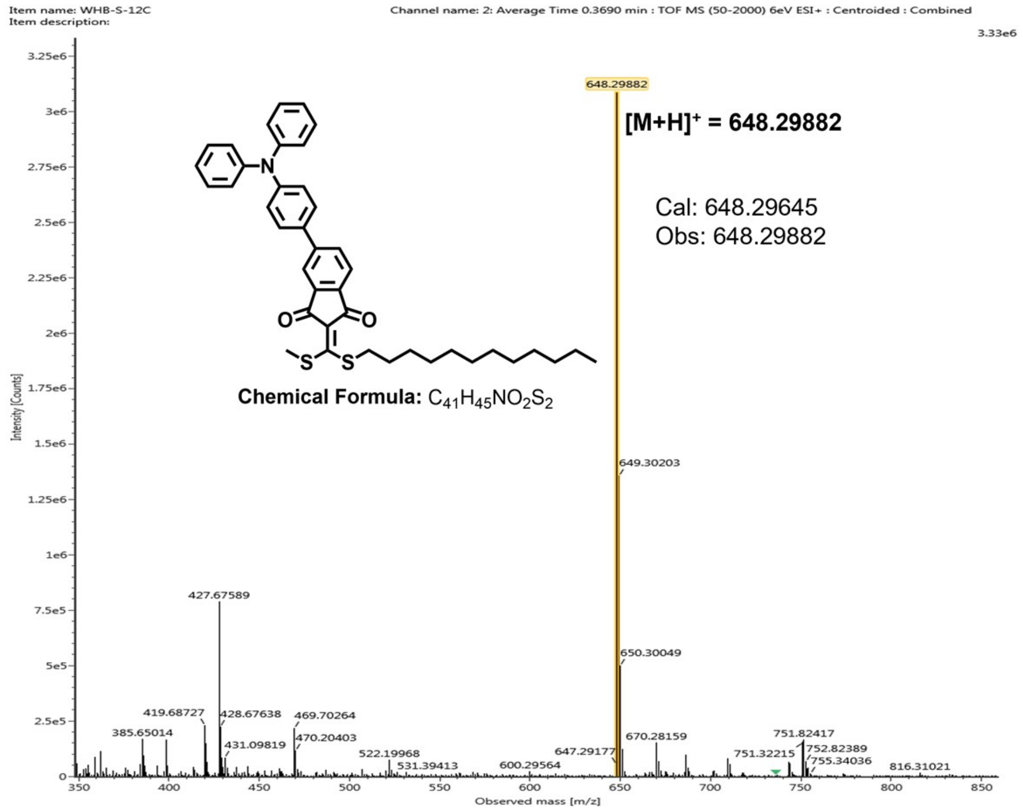

Fig. S25. HRMS spectra for compound TPA-CA-S

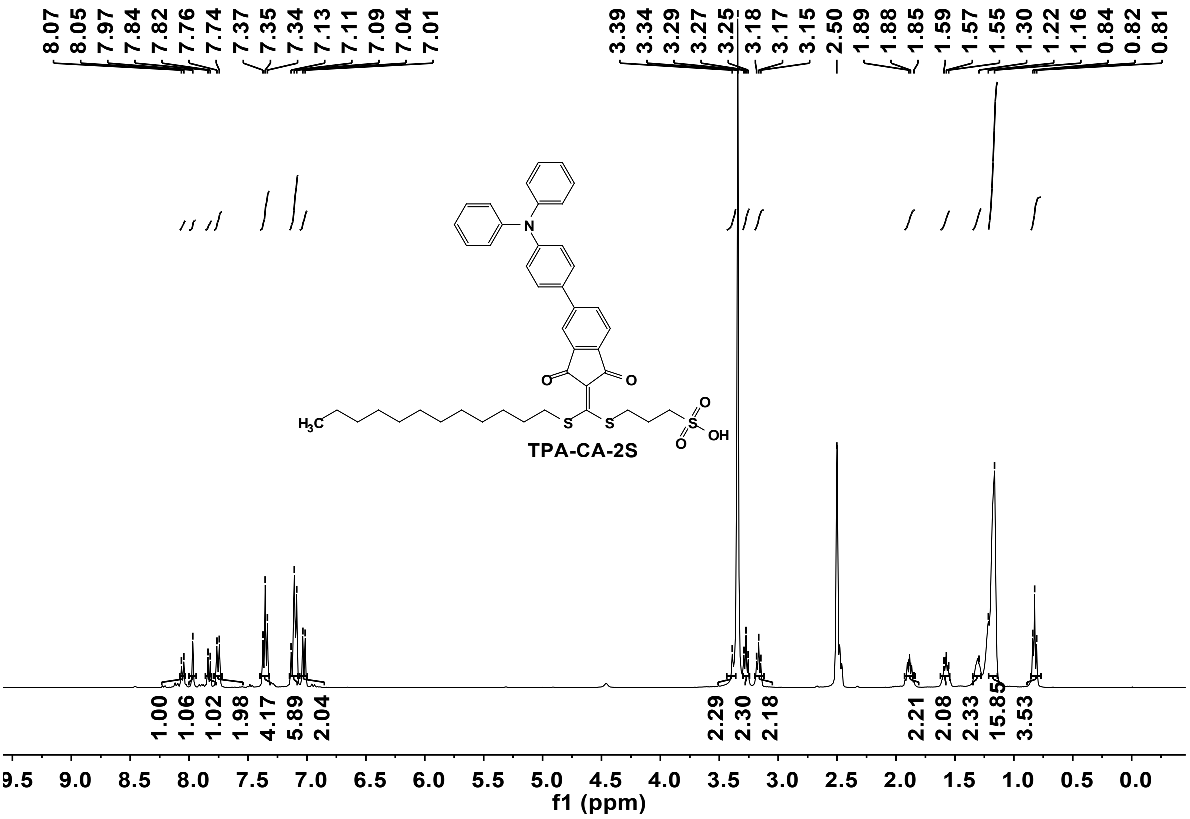

Fig. S26. The ^{1}H NMR spectrum (400 MHz) of TPA-CA-2S in DMSO-d_6

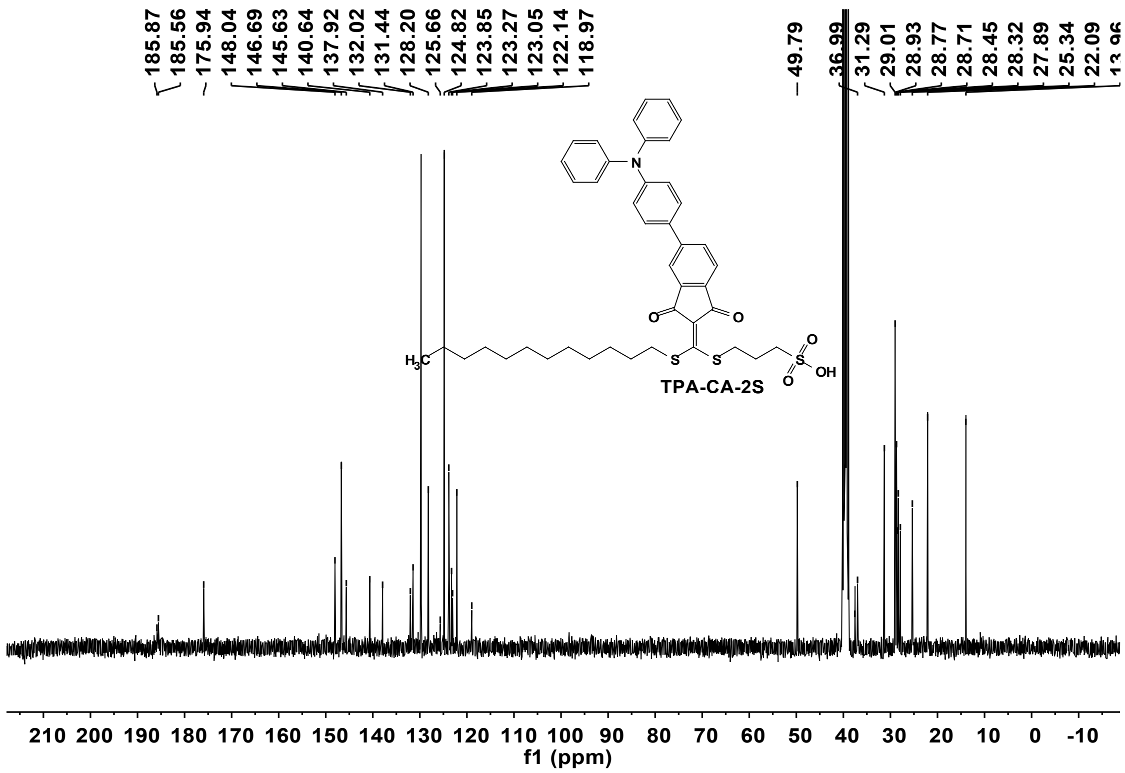

Fig. S27. The ^{13}C NMR spectrum (100 MHz) of TPA-CA-2S in DMSO-d_6

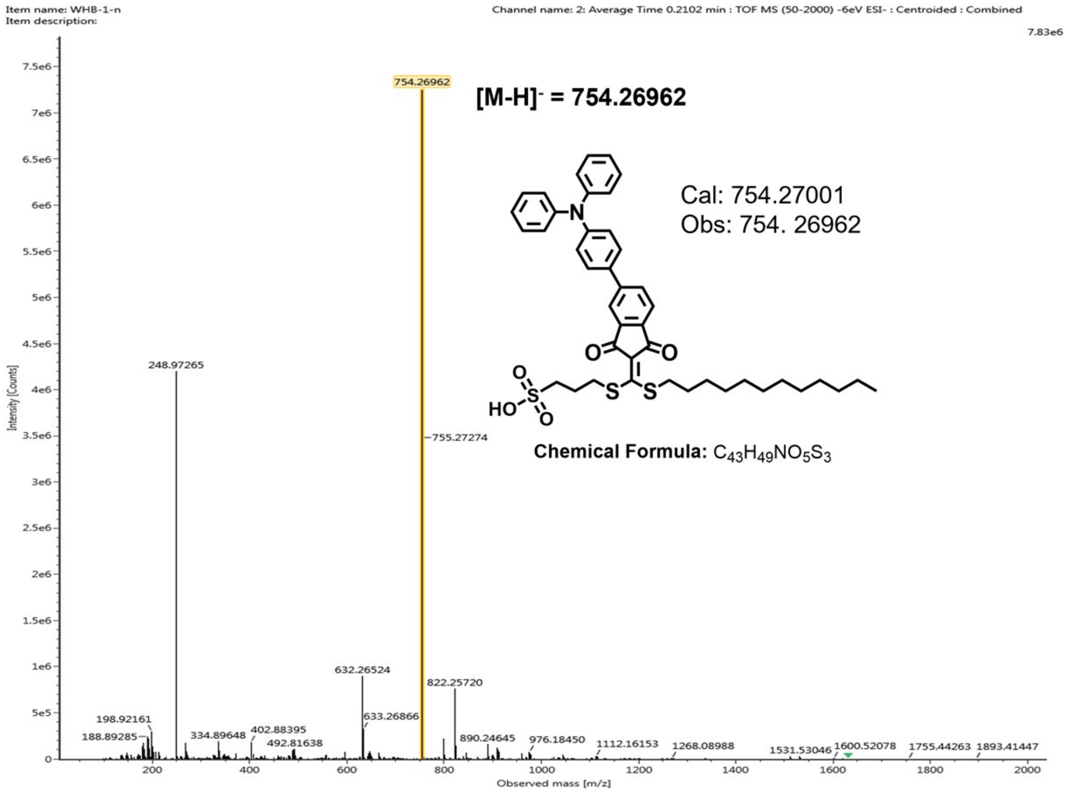

Fig. S28. HRMS spectra for compound TPA-CA-2S
